# CRISPR/Cas9-mediated mutagenesis of phytoene desaturase in diploid and octoploid strawberry

**DOI:** 10.1186/s13007-019-0428-6

**Published:** 2019-05-02

**Authors:** Fiona M. Wilson, Kate Harrison, Andrew D. Armitage, Andrew J. Simkin, Richard J. Harrison

**Affiliations:** 1 NIAB EMR, New Road, East Malling, Kent ME19 6BJ UK; 2 Present Address: Driscolls, New Road, East Malling, Kent ME19 6BJ UK

**Keywords:** *Fragaria*, Strawberry, Diploid, Polyploid, CRISPR/Cas9, Phytoene desaturase

## Abstract

**Background:**

Gene editing using CRISPR/Cas9 is a simple and powerful tool for elucidating genetic controls and for crop improvement and its use has been reported in a growing number of important food crops, including recently *Fragaria*. In order to inform application of the technology in *Fragaria, w*e targeted the visible endogenous marker gene *PDS* (phytoene desaturase) in diploid *Fragaria vesca* ssp. *vesca* ‘Hawaii 4’ and octoploid *F.* × *ananassa* ‘Calypso’.

**Results:**

*Agrobacterium*-mediated transformation of leaf and petiole explants was used for efficient stable integration of constructs expressing plant codon-optimised Cas9 and single guide sequences under control of the *Arabidopsis U6*-*26* consensus promoter and terminator or *Fragaria vesca U6III* regulatory sequences. More than 80% (‘Hawaii 4’) and 50% (‘Calypso’) putative transgenic shoot lines (multiple shoots derived from a single callus) exhibited mutant phenotypes. Of mutant shoot lines selected for molecular analysis, approximately 75% (‘Hawaii 4’) and 55% (‘Calypso’) included albino regenerants with bi-allelic target sequence variants. Our results indicate the *PDS* gene is functionally diploid in ‘Calypso’.

**Conclusion:**

We demonstrate that CRISPR/Cas9 may be used to generate biallelic mutants at high frequency within the genomes of diploid and octoploid strawberry. The methodology, observations and comprehensive data set presented will facilitate routine application of this technology in *Fragaria* to single and multiple gene copy targets where mutant phenotypes cannot be identified visually.

**Electronic supplementary material:**

The online version of this article (10.1186/s13007-019-0428-6) contains supplementary material, which is available to authorized users.

## Background

The cultivated strawberry, *Fragaria* × *ananassa*, is one of the most important fruit crops in the family Rosaceae. It is among the most widely grown and consumed fruit throughout the world, with global production reaching 9.1 M tonnes in 2016, an increase over the previous decade of more than 5% annually (www.freshfruitportal.com). In the UK alone, in 2016 120,000 tonnes were produced, with a market value of £260 million [1].

Traditional breeding is lengthy and difficult as *F.* × *ananassa* is an octoploid species with a complex genome, and is intolerant to inbreeding. Important for genetic improvement is the potential to enhance elite cultivars using genetic modification, first demonstrated with marker genes using *Agrobacterium*-mediated transformation in the 1990s [2, 3]. Since then a number of developments in transformation methodology and its application in *Fragaria* have been reported [4, 5]. The diploid wild strawberry *F. vesca* shares a high degree of sequence identity with the cultivated strawberry and is a model for genetic improvement in the genus due to its small genome size (240 mb), short generation time, and ease of genetic transformation [6, 7]. The availability of the complete genome for *F. vesca* [8, 9] and chromosome-scale genome assembly of the octoploid genome [10] facilitates the identification and manipulation of genes controlling important traits.

From its first use in model species such as *Arabidopsis* [11, 12] and tobacco [12, 13] the rapid development of CRISPR/Cas9 technology has enabled widespread use of precise gene editing in plants [14–16]. Reports of successful gene editing in diploid crops include rice [17, 18], sorghum [19], tomato [20], maize [21, 22], *Populus* [23], apple [24], grapes [25] and cassava [26, 27]. In polyploid crops, multiple gene homoeoalleles have been simultaneously edited using CRISPR/Cas9 in wheat [28], potato [29] and *Brassica napus* [30, 31]. Multiple gene targets have been successfully targeted in Arabidopsis [32], maize [33] and rice [34, 35]. Recently, successful use of CRISPR/Cas9 has been reported for elucidation of plant and fruit development in diploid and octoploid *Fragaria* [36, 37].

Defects in phytoene desaturase (*PDS)* gene function result in a distinctive albino phenotype [38], and recent studies in *Populus* [23], apple [24], grapes [25] and cassava [26] have targeted the *PDS* gene to demonstrate successful genome editing in these crops. Nishitani et al. [24] compared target sites in different exons of the apple *PDS* gene and showed that the greatest mutation efficiency was achieved by targeting exon 7. As strawberry is also a member of the family *Rosaceae*, we chose to target the same region in the *PDS* gene of diploid and octoploid strawberry.

Our results show the mutation of *PDS* in diploid and octoploid strawberry results in a clear albino phenotype at a high frequency. By providing a detailed phenotypic and molecular analysis of CRISPR/Cas9 editing of a marker gene in transgenic populations of both diploid and octoploid *Fragaria*, this work complements recently reported studies targeting developmental genes in *Fragaria* [36, 37]. The methodology and data provided will inform future application of CRISPR/Cas9 technology in *Fragaria*, both as a research tool and for modifying molecular mechanisms controlling traits of agronomic importance, where mutant phenotypes cannot be identified visually.

## Results and discussion

### Transformation and regeneration of mutant lines

**Fig. 1:**
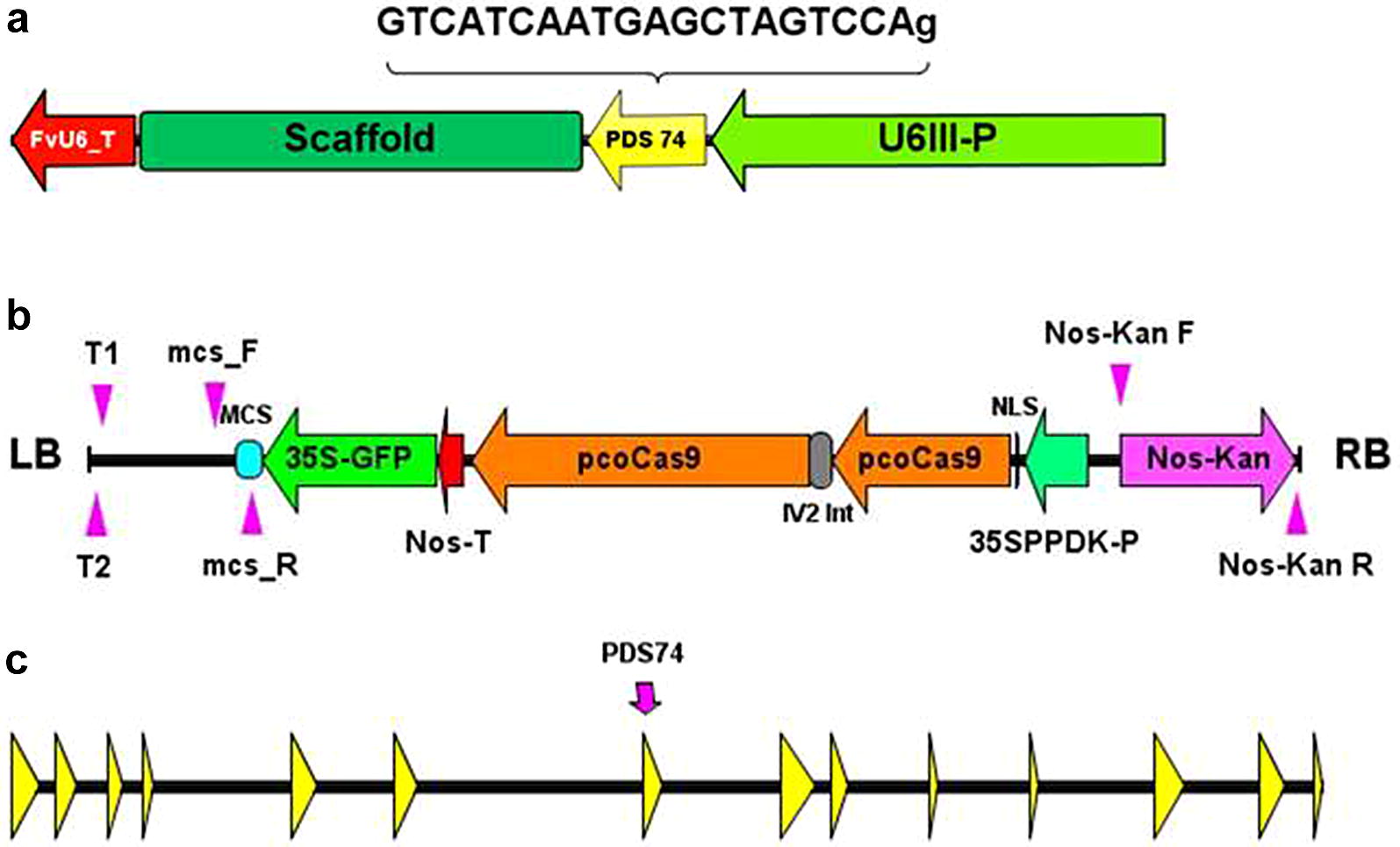
Schematic maps of CRISPR cassette, T-DNA of pCas9-K-GFP and *Fragaria vesca PDS* gene. **a** Single guide RNA inserted into the multiple cloning site (MCS) of pCas9-K-GFP. **b** pCas9-K-GFP T-DNA showing the relative position between the left border (LB) and right border (RB) of the MCS, the marker/selection expression cassettes 35S-GFP and Nos-Kan and the pcoCas9 coding sequence with the potato IV2 intron (IV2 Int), nuclear localistion signal (NLS), 35SPPDK promoter and Nos terminator. The binding sites  of primers used for TAIL-PCR (T1 = Tail 1; T2 = Tail 2), sgRNA insertion site PCR (mcs_F; mcs_R) and Nos-Kan cassette PCR (Nos-Kan F; Nos-Kan R) are indicated. **c** *Fragaria vesca PDS* gene, LG4-gene12690 (8035 bp, FvAssembly v4) showing exons (yellow arrows) and the location of the CRISPR target sequence PDS74 (position 3’–5’ = 3873–3892)

We placed sgRNAs targeting exon 7 of the *PDS* gene under transcriptional control of the *Arabidopsis U6*-*26* promoter (p*AtU6*-*26* vector) [13] or the corresponding sequence of the *F. vesca U6III* promoter (p*FvU6III* vector) (Fig. 1, Additional file 1: Table S1).

Following transformation, calli were maintained on selective regeneration medium for up to 7 months and pieces of callus with shoots were successively harvested as shoots regenerated. Following the first passage on selective medium, calli were transferred to regeneration medium with or without GA_3_ as it was unknown whether mutation of the *PDS* gene would inhibit gibberellic acid synthesis and consequently affect shoot regeneration. Mutant shoots were efficiently obtained on medium without GA_3_ and there is no clear indication of the effect of including GA_3_ in the regeneration medium (Table 1). On average, approximately 80% of ‘Hawaii 4’ and ‘Calypso’ shoot lines (shoots derived from single calli) formed roots on kanamycin or exhibited clear mutant phenotypes (Table 1).

**Table 1:** Numbers and percentages of ‘Hawaii 4’ and ‘Calypso’ shoot lines regenerating on selective medium

Promoter	Regeneration medium ± GA_3_	Number of lines harvested throughout the experiment				Total lines harvested	Number of lines assessed for transformed phenotype	Putative transformed lines	% Putative transformed lines	Lines with mutant phenotype	% Putative transformants with mutant phenotype	Cultivar
		2 months	4 months	5 months	7 months							
At	+ GA	18	18	22	7	65	58	42	72	24	57	Hawaii-4
At	no GA	1	4	1	0	6	6	5	83	1	20	
Fv	+ GA	12	1	39	4	56	55	50	91	46	92	
Fv	no GA	15	8	15	0	38	37	26	70	21	81	
Average									79		62	
At	+ GA	0	11	10	21	42	30	22	73	8	36	Calypso
At	no GA	0	9	7	6	22	19	16	84	11	69	
Fv	+ GA	2	4	1	6	13	9	8	89	4	50	
Fv	no GA	2	3	4	3	12	10	8	80	2	25	
Average									82		45	

Explants were regenerated on selective medium with (+) or without (no) GA_3_, following transformation with sgRNAs under transcriptional control of the *Arabidopsis* (At) or the *F. vesca* (Fv) *U6III* promoters. ‘Numbers of lines harvested throughout the experiment’ are callus lines initiating shoots at 2, 4, 5 and 7 months. ‘Putative transformed lines’ are lines either forming roots on medium supplemented with kanamycin and/or regenerating shoots with a mutant phenotype

Sixty-two percent of ‘Hawaii 4’ and 45% of ‘Calypso’ putative transgenic shoot lines exhibited mutant phenotypes (Table 1). Where shoot lines comprised a range of phenotypes, albino or variegated phenotypes were apparent in approximately 10% of tissues harvested. Mutant phenotypes were evident on regeneration plates and also became apparent after calli were transferred to rooting medium. Most calli regenerated a mixture of mutant phenotypes, as described within this text and in supplementary files (Additional file 2: Figure S1, Additional file 3: Figure S2, Additional file 4: Table S2, Additional file 5: Table S3). Albino shoots regenerated either directly from callus, or as axillary shoots derived from variegated shoots. The proportions of ‘Hawaii 4’ and ‘Calypso’ mutant phenotypes derived from p*AtU6*-*26* are 25/47 lines (53%) and 19/38 lines (50%), respectively, and corresponding data for p*FvU6III* are 67/76 lines (88%) and 6/16 lines (38%). Native promoters are known to enhance mutation efficiency: for example, in soybean in three target genes, mutation efficiencies were increased by the use of the soybean *U6-10* promoter compared to the *Arabidopsis* *U6-26* promoter [39]. Relative mutation efficiencies in this experiment suggest that the *F. vesca U6III* promoter may be more effective than the *Arabidopsis U6*-*26* consensus promoter in ‘Hawaii 4’ but not in ‘Calypso’, and that alternative *U6* promoters native to the octoploid genome may enhance sgRNA expression in ‘Calypso’ and other commercial cultivars.

### Types and frequency of target site mutations in transgenic lines

Amplicons of 401 bp spanning the target site were generated for 96 leaf samples (Additional file 6: Fig. S3) and used for Illumina MiSeq sequencing. Sequence data was obtained for a range of phenotypes from single and multiple shoots of 19 ‘Hawaii 4’ and 8 ‘Calypso’ shoot lines (criteria for selection were phenotype and availability of suitable material at the time of analysis) and for a single wild-type (WT) shoot of each cultivar regenerated from in vitro explants.

**Fig. 2:**
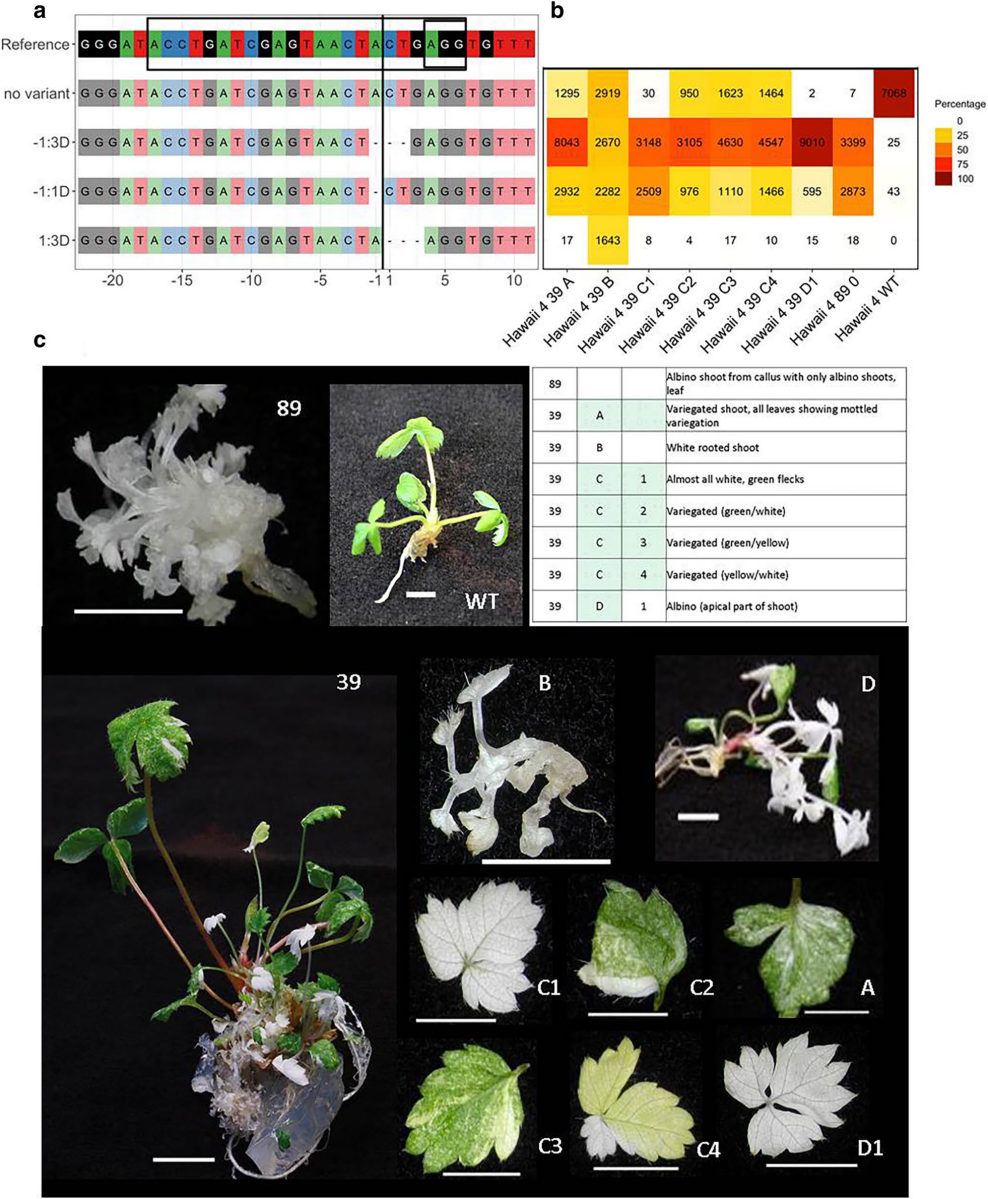
Sequence mutations and corresponding phenotypes of CRISPR/Cas9 transgenic lines of ‘Hawaii 4’. Examples of sequence mutations and allelic variants with corresponding phenotypes in transgenic shoot lines of *Fragaria* ‘Hawaii 4’ (line numbers 39 and 89). **a** Alignment of the sequence for each variant to the reference sequence. To the left of the panel, each sequence variant type is identified by the location of the mutation relative to the cut site:number bases deleted (D). Deletions result in amino acid loss (mutations − 1:3D and 1:3D), and also frameshift (mutation − 1:1D) and generation of stop codons within exon 7 resulting in truncated PDS protein. The cut site is shown by a black vertical line; the target site and PAM site (AGG) are within the box in the reference sequence. **b** Heat map showing the number and percentage of variant reads for each sample. Samples are arranged in columns and identified below the panel. **c** Images of phenotypes and an accompanying table giving descriptions for each sequenced sample. Numbers in the first column refer to the shoot line. In subsequent columns, individual shoots from a shoot line are designated using a letter (A, B, etc.) and numbers indicate individual leaves from individual shoots. White- or pale-green-box shading indicates albino or pale green/variegated phenotype, respectively, for shoots and leaves. WT = wild type. Scale bars are 5 mm

**Fig. 3:**
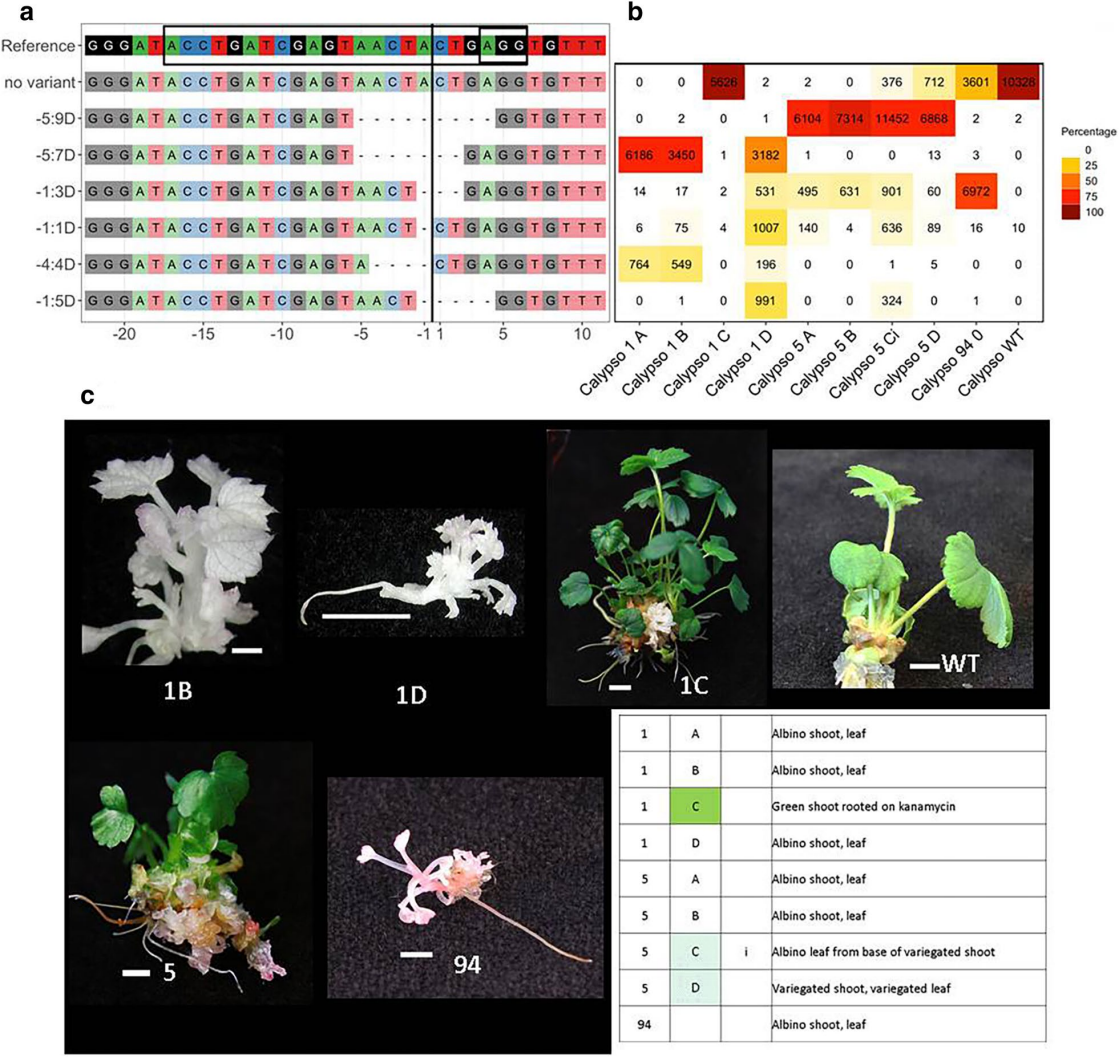
Sequence mutations and corresponding phenotypes of CRISPR/Cas9 transgenic lines of ‘Calypso’. Examples of sequence mutations and allelic variants with corresponding phenotypes in transgenic shoot lines of *Fragaria* ‘Calypso’ (line numbers 1, 5 and 94). **a** Alignment of the sequence for each variant to the reference sequence. To the left of the panel, each sequence variant type is identified by the location of the mutation relative to the cut site:number of bases deleted (D). Deletions result in amino acid loss (mutations − 1:3D and − 5:9D), frameshift (mutation − 1:1D, − 1:5D, − 4:4D and − 5:7D) and generation of stop codons within exon 7 resulting in truncated PDS protein. The cut site is shown by a black vertical line; the target site and PAM site (AGG) are within the box in the reference sequence. **b** Heat map showing the number and percentage of variant reads for each sample. Samples are arranged in columns and identified below the panel. **c** Images of phenotypes and an accompanying table giving descriptions for each sequenced sample. Numbers in the first column refer to the shoot line. In subsequent columns individual shoots from a shoot line are designated using a letter (A, B, etc.) and numbers indicate individual leaves from individual shoots. White-, pale-green- or dark-green-box shading indicates albino, pale green/variegated or green phenotype respectively, for shoots and leaves. WT = wild type. Scale bars are 5 mm

Transgenic shoot lines exhibit a variety of mutant sequence variants at and around the target site in a highly conserved region of the *PDS* gene resulting in phenotypes typical of defective *PDS* gene function, including pale green, variegated and albino phenotypes, which are often (although not always, as discussed below) related to the proportion of variant reads seen (Figs. 2, 3, Additional file 2: Fig. S1, Additional file 3: Fig. S2, Additional file 4: Table S2). Approximately 60–80% shoot lines included albino shoots with 100% target site sequence variant reads.

The most common sequence mutations are three variant types with 1, 2 or 3 base deletions at (or spanning) the cut site in exon 7 of the *PDS* gene (Table 2, Additional file 7: Fig. S4): all are seen in the majority of ‘Hawaii 4’ lines and about half of ‘Calypso’ lines. Types and frequency of sequence mutations are summarised in Table 2 and illustrated in Figs. 2, 3, Additional file 2: Fig. S1 and Additional file 3: Fig. S2. Protein translation of in-frame mutations (deletions of 3, or multiples of 3, bases) shows loss of one or more amino acids around the cut site (Additional file 7: Fig. S4). Frameshift mutations (and mutation − 44:45D) result in amino acid loss and generation of stop codons within exon 7, resulting in truncated PDS protein.

**Table 2:** Types and frequency of sequence mutation in exon 7 of the *PDS* gene in *Fragaria*

Sequence variant	Hawaii 4	Calypso	Non-synonymous amino acid substitution	Frameshift and protein truncation	Amino acid deletion	Putative functional protein
− 1:1D	17	4	–	+	+	–
− 2:1D	2	–	–	+	+	–
− 2:2D	10	3	–	+	+	–
− 1:3D	11	3	–	–	+	–
− 4:4D	–	1	–	+	+	–
− 1:5D	2	3	–	+	+	–
− 2:6D	1	–	–	–	+	–
− 3:7D	–	1	–	+	+	–
− 5:7D	–	1	–	+	+	–
− 2:9D	1	–	–	–	+	–
− 5:9D	–	1	–	–	+	–
− 6:9D	1	–	–	–	+	–
− 10:9D	1	–	–	–	+	–
− 11:15D	5	–	–	–	+	–
− 1:22D	1	–	–	+	+	–
− 25:27D	2	–	–	–	+	–
− 44:45D	1	–	–	+	+	–
1:3D	2	–	–	–	+	–
1:5D	1	–	–	+	+	–
1:1I	3	–	+	+	–	–
SNV:− 3A and 1:2	1	–	+	+	–	–
SNV:1A	1	–	+	–	–	+

The numbers of shoot lines with each variant type are shown for CRISPR/Cas9 transgenic lines of ‘Hawaii 4’ and ‘Calypso’. Each sequence variant type is identified by the location of the mutation relative to the cut site:number bases deleted (D), inserted (I), or substituted (SNV:location relative to cut site and base substitution). The resultant amino acid changes and consequences for PDS protein function for each sequence variant type are indicated by ‘+’ or ‘−’. Amino acid substitutions (generated by insertions or base substitutions) are non-synonymous

### Types of allelic variant and transgenic origin of multiple shoots from individual callus lines

Leaf samples from individual shoots are either homozygous for one sequence variant type, heterozygous (having two sequence variant types), chimaeric (3 or more sequence variant types), or have a mixture of wild-type sequence and 1, 2 or more than 2 sequence variants: mono-WT, di-WT or multi-WT (Figs. 2, 3, Additional file 2: Fig. S1, Additional file 3: Fig. S2, Additional file 4: Table S2). Most homozygous and heterozygous allelic variants are of the most common sequence variant types and may have arisen from simultaneous mutation of both alleles prior to division of the cell from which the tissue is derived or at a subsequent stage in organogenesis. Multiple samples from single shoots and shoot lines often share variant sequence types and combinations of sequence types (Additional file 4: Table S2, Figs. 2, 3, Additional file 2: Fig. S1, Additional file 3: Fig. S2), including more complex patterns of chimaeric allelic variation. The timing and sequence of mutation events will determine the mixture of the allelic variants within a shoot and shoot line. The data for multiple shoots from shoot lines and leaf samples from single shoots suggest albino shoots regenerate from callus (e.g. ‘Hawaii 4’ 89, ‘Calypso’ 1, Figs. 2, 3, Additional file 5: Table S3) or by re-arrangement of existing mutations and/or continued gene editing as apical variants or axillary branches from variegated shoots (e.g. ‘Hawaii 4’ 39D Fig. 2, Additional file 5: Table S3). Analysis of leaves of some shoots provides evidence of ongoing gene editing within a shoot (e.g. ‘Hawaii 4’ 2C, 28C, 39C, D, 46Dii, H, ‘Calypso’ 5C, Figs. 2, 3, Additional file 2: Fig. S1, Additional file 4: Table S2, Additional file 5: Table S3). Four of 7 mutant ‘Calypso’ shoot lines (lines 1, 5, 7, 100) and 15 of 19 mutant ‘Hawaii 4’ shoot lines (all lines except 9, 10, 32, and 36) produced shoots which are biallelic (homozygous, heterozygous or chimaeric) mutants.

To aid interpretation of the patterns of allelic variation, TAIL PCR was performed to generate data relating to T-DNA integration (Additional file 4: Table S2, Additional file 8: Fig. S5). Multiple shoots from individual lines were included and putative plant chromosomal sequence (i.e. not matching vector sequence) at the left border integration site, often contiguous with partial or complete T-DNA left border sequence, was recovered from approximately half of shoot lines screened (7 of 17 ‘Hawaii 4’ and 5 of 8 ‘Calypso’ lines, Additional file 9: Fig. S6, Additional file 4: Table S2). In all cases a single unique sequence for individual lines was obtained (Additional file 10: Table S4) and where integration data was obtained for multiple shoots the data was homologous. For the remainder of shoot lines the sequence obtained beyond the T-DNA left border matches only binary vector sequence (Additional file 9: Fig. S6), indicating chromosomal integration of vector ‘backbone’ DNA, which was shown by De Buck et al. [40] to be a common occurrence in transgenic *Arabidopsis* and tobacco plants. Shoots of ‘Hawaii 4’ line 10 have a complex integration sequence comprising T-DNA, vector backbone sequence and sequence not matching vector DNA (Additional file 9: Fig. S6).

The combined data from both target site sequence analysis and TAIL PCR sequence analysis indicate that multiple shoots regenerated from individual callus lines are likely to share a transgenic integration origin. The only shoot line where the data indicate separate transgenic origin of individual shoots is ‘Hawaii 4’ line 79: 79C and 79E TAIL sequences do not overlap but both align to the vector backbone and may be the same (Additional file 9: Fig. S6); but 79A has a more complex pattern of mutation and does not align to vector sequence.

### Correlating observed phenotypes with target site mutation data

Phenotypic observations of leaf samples of multiple shoots were compared to the mutant sequence data generated for each sample (Figs. 2, 3, Additional file 2: Fig. S1, Additional file 3: Fig. S2, Additional file 4: Table S2) and to WT sequence data. All WT shoots regenerated in these experiments on non-selective medium were observed to be green (i.e. no albino or variegated phenotypes were observed), which is consistent with observations made in previous transformation work. The clear albino and variegated phenotypes seen are quite distinct from the bleaching of untransformed tissue cultured on kanamycin, and these are therefore attributed to *PDS* gene mutation. All transformed shoot lines comprised a mixture of green, and/or variegated, pale green, or albino shoots, except ‘Hawaii 4’ line 89 (Fig. 2), from which only albino callus and shoots regenerated. A very small fraction of variant reads was identified in sequence data from wild-type samples: 0.6% and 0.02% of total reads in the data for ‘Hawaii 4’ and ‘Calypso’ wild types, respectively. These reads were considered background ‘noise’ as a result of index-switching between Illumina barcodes, an event that occurs at low rates during Illumina sequencing of pooled samples on a single flow cell [41].

In most cases the observed phenotype reflects the sequence data obtained. Sequence data for the majority of albino samples in both cultivars lack or have a negligible fraction of wild-type sequence reads (< 0.05–0.1% of total reads). For some shoot lines the fraction of wild-type sequence data for all shoots sequenced is negligible (‘Hawaii 4’ lines 12, 14, 79 and 89 and ‘Calypso’ line 1). The exception is ‘Calypso’ shoot 1C, which is green and probably not transformed (PCR data show the absence of a product in reactions using Nos-Kan cassette and sgRNA insert site T-DNA primers). Sequence data for ‘Calypso’ shoot line 7D (Additional file 3: Fig. S2) is anomalous as it was observed to have a variegated phenotype yet WT sequence reads were not obtained. The presence of a low fraction (< 5%) of wild-type reads in data from white phenotypes in some cases (Additional file 4: Table S2, Additional file 5: Table S3) may be attributed to difficulty in assessing phenotype (for example where the leaf samples are very small, e.g. ‘Hawaii 4’ 39B, ‘Calypso’ 5 Ci, Figs. 2, 3, Additional file 5: Table S3).

‘Hawaii 4’ samples, which are largely white with some green and a high fraction of wild-type reads e.g. 28 D1, 32 B1, B2 and 36 A3 (Additional file 2: Fig. S1), are possibly chimaeras with a multiple transgenic origin: for example, a mosaic of white and green cells in the L2 layer would be manifested as small green flecks at the leaf edge, and would explain the high wild-type reads in comparison with samples that show small green sectors in the central part of the leaf lamina (e.g. ‘Hawaii 4’ 2 Ci and 39 C1, with < 0.7% wild-type reads). Regeneration of chimaeric shoots comprised of transgenic and non-transgenic cells has been documented in transformed strawberry [2] and its frequency can be minimised by employing a stringent selection method (as described in this study). Chimaeric origin of shoots from more than one transformed cell is possible; however Southern blot data reported for diploid and octoploid strawberry [7, 42, 43], indicate that transformants will usually have a single copy (or few T-DNA copies with bands of similar intensity) and by inference these will be of single cell origin. Direct organogenesis (omitting a callus phase) would avoid regeneration of chimaeric shoots, but the potential for this is cultivar dependent [44].

Only one transgenic shoot 100% heterozygous for a target sequence mutation lacks the expected albino phenotype: sequence data for a green ‘Hawaii 4’ transgenic shoot, ‘Hawaii 4’ 3A (Additional file 2: Fig. S1), show that 50% of reads are deletion variants, but indicate that one allele has an amino acid substitution (threonine to asparagine) and that this encodes a functional PDS protein. Published PDS protein sequences for maize and rice also encode asparagine at this position [45] (Additional file 11: Fig. S7).

The ranges of wild-type reads commonly observed for pale green and variegated samples are 7–19% (seen in 7 samples from 7 lines of ‘Hawaii 4’) and 6–36% (seen in 15 samples from 10 lines of ‘Hawaii 4’ and 2 samples from 2 lines of ‘Calypso’), which are taken to be due to the proportion of alleles and cells expressing functional PDS protein.

In lines of both cultivars, sequence data for some phenotypes show unexpected proportions of wild-type and mutated sequences (Additional file 4: Table S2, Additional file 5: Table S3, Additional file 2: Fig. S1, Additional file 3: Fig. S2). All ‘Hawaii 4’ transgenic samples with a dark green phenotype (apart from 3A which has a sequence mutation that encodes a functional PDS protein) are single-allele sequence variants with only approximately 20% to 60% wild-type reads. In addition, there are white phenotypes with higher than expected wild-type sequence fractions (15–30%). Sequence data for ‘Calypso’ albino shoot 94 (Fig. 3) include 31% wild-type reads and a green transgenic shoot (‘Calypso’ 59, Additional file 3: Fig. S2) has only 11% wild-type reads. In apple (another diploid member of the Rosaceae), Nishitani et al. [24] also observed green transformants with partially mutated sequences of the *PDS* gene. It is possible that one of the *PDS* alleles in *F. vesca* is non-functional and that in the octoploid, cultivated strawberry there may be only one functional *PDS* gene allele in which case the fraction of wild-type sequence would reflect the proportion of non-functional *PDS* alleles. Albino or variegated samples with unexpectedly high wild-type reads and complex chimaeric genotypes may include mutations of both functional and non-functional alleles (Additional file 5: Table S3). Evidence for gene silencing following gene duplication and loss of redundant sequences in polyploids is well-documented [46], and gene copy loss and deletion in polyploid *Fragaria* has been described by Rousseau-Gueutin et al. [47]. The fact that a single wild-type amplicon was found is suggestive, but not conclusive, that the *PDS1* locus is functionally diploid and that complex variants observed in amplicon profiles are due to chimaeric regeneration rather than multiple edits of homeologs.

## Conclusion

The primary aim of this work was to generate a set of detailed data using a visible marker that will be of relevance for gene editing in *Fragaria* of single- and multi-copy genes for which there is no visible phenotype. We have shown that via *Agrobacterium*-mediated transformation of petioles and leaves it is possible to regenerate shoots that are biallelic mutants for a single copy gene at a high frequency in both the diploid and octoploid genomes, providing evidence that effective targeting of multiple alleles and gene copies should be routinely achievable in *Fragaria*. It is possible that use of alternative *U6III* promoters may enhance expression of guide sequences and increase mutagenesis in octoploid genomes. Our data corroborate and complement recent reports describing CRISPR/Cas9 mutagenesis in *Fragaria*: similar rates of mutation have been reported by Zhou et al. [36] in wild strawberry and they demonstrate improved recovery of mutants by use of dual sgRNAs. Effective targeting of multiple homoeologs in octoploid strawberry has been demonstrated using dual sgRNAs by Martín-Pizarro et al. [37]. We provide further, comprehensive data, of relevance to both single and multiple gene copy targets, detailing the frequency and occurrence of mutant phenotypes and corresponding target sequence mutations. Our observations indicate continued gene editing (this could be substantiated by assessing *Cas9* and guide transcript expression in variegated shoots) and/or re-arrangement of existing mutations during organogenesis and shoot development and support the expectation that the frequency of biallelic mutants will increase with continued propagation, which is of particular interest for editing multiple gene copies in cultivated strawberry.

For potential non-research applications of gene editing technology in octoploid cultivars (for which removal of T-DNA integrations by crossing is not a feasible option), strategies to avoid T-DNA integration might include maximising transient expression from the binary vector, inhibiting T-DNA integration and selection against stably transformed cells. Use of non-DNA technologies such as CRISPR-Cas9 ribonucleoprotein complexes (RNPs) should also be considered: efficient use of RNPs has been demonstrated in some crops such as potato [48] and Brassica [49], and its use in *Fragaria* would be dependent upon the ability to regenerate shoots from protoplasts [50].

In summary, our detailed study describing CRISPR/Cas mutagenesis of a visible marker, when combined with high-throughput screening [51] and high-throughput sequencing, is expected to facilitate the routine, efficient generation of single and multi-copy gene mutants in both diploid and octoploid *Fragaria*.

## Methods

### Construction of CRISPR/Cas9 vectors

The binary vector pFGC-pcoCas9 (Addgene plasmid # 52256) was modified by insertion of pGreen plant marker constructs [52]: the ‘Nos-Kan’ cassette (comprising the *nptII* gene under control of nopaline synthase regulatory sequences) was cloned into the *Pme*1 site near the right border and the ‘35S-GFP’ cassette (comprising the modified *GFP* gene [53] under control of *35S* regulatory sequences) was cloned into the *Sma*1 site in the multiple cloning site to give pCas9-K-GFP (Fig. 1). The GFP marker was not used in this study.

CRISPR cassettes incorporating the sgRNA sequence and regulatory elements (Fig. 1) were synthesised by Integrated DNA Technologies (IDT) Ltd and cloned into pCR^®^-Blunt II-TOPO^®^ (Thermo Fisher Scientific, UK). Cassettes were inserted into the *Eco*R1 site of pCas9-K-GFP and the orientation confirmed by sequencing. The *PDS* coding sequence for *Malus domestica* (GenBank: KU508828.1) was BLAST aligned against the genomes for *F. vesca* (FvAssembly 1.1) and *F.* × *ananassa* (www.rosaceae.org). The CRISPR target sequence selected (ACCTGATCGAGTAACTACTGAGG) is common to both *Fragaria* genomes and is located on the sense strand in exon 7 of FvAssembly 4 [9] (Fig. 1). It corresponds almost exactly to the target sequence (ex7-20 bp, ACCTGATCGAGTAACTACAGAGG) used successfully by Nishitani et al. [24]. The PAM sequences are underlined. The target guide RNA (gRNA) sequence (PDS74) comprises the first 20 base pairs of the target sequence preceded by an additional guanine base to maintain the native *U6* promoter start of transcription. This is placed under transcriptional control of the consensus sequence of the *Arabidopsis U6*-*26* promoter [13] or the corresponding sequence of the *F. vesca U6III* promoter and terminator sequences (Additional file 1: Table S1).

### In vitro culture of *Fragaria* stock cultures

In vitro shoot cultures of *F. vesca* ‘Hawaii 4’ and *F.* × *ananassa* ‘Calypso’ were maintained in a growth room at 20 °C with a 16/8 h light/dark photoperiod provided by fluorescent lamps (colour reference 835, colour temperature 3500 K). Crowns were sub cultured at 4–5 week intervals, 5 per honey jar containing 50 ml medium. Basal culture medium was Murashige and Skoog (MS) macro and micro elements and vitamins [54], supplemented with sucrose (30 g l^−1^) and solidified with Daishin agar (Duchefa D1004, 9 g l^−1^). The pH was adjusted to 5.8 before autoclaving. Shoots were alternately cultured on basal medium supplemented with 6-benzylaminopurine (BAP) 0.1 mg l^−1^ and indole-3-butyric acid (IBA) 0.1 mg l^−1^ or BAP 0.5 mg l^−1^, as described by Schaart [55].

### Transformation of diploid and octoploid strawberry

Transformation of ‘Calypso’ was performed essentially as Schaart [55], with minor modifications. The protocol was additionally modified to achieve efficient transformation of ‘Hawaii 4’ by use of petiole explants in place of leaf explants and initial culture in liquid selection medium, as described below. *Agrobacterium tumefaciens* strain EHA105 [56] harbouring the binary vector was grown overnight, and pelleted at 2000×*g* for 10 min. The inoculum was prepared by re-suspending the overnight culture in filter-sterilised liquid MS-based medium supplemented with glucose (30 g l^−1^) and acetosyringone (100 µM), pH 5.2, to give OD 600 nm 0.2–0.3. Petioles from apical leaves of ‘Hawaii 4’ or leaflets from young expanding leaves of ‘Calypso’ were harvested from shoots 4 weeks after subculture and submerged in the inoculum for approximately 10–15 min during explant preparation: petioles were cut into 4–5 mm pieces and leaflets were separated from each leaf before scoring transversely into 2 mm strips, leaving one leaf edge intact. Explants were then blotted to remove excess inoculum before transfer to Shoot Regeneration Medium (SRM): MS medium supplemented with α-naphthaleneacetic acid (NAA) (0.2 mg l^−1^) and thidiazuron (TDZ) (1 mg l^−1^). The pH was adjusted to 5.8 and Agargel ™ (A3301, Sigma) (5 g l^−1^) was added before autoclaving. After autoclaving filter-sterilised glucose (300 g l^−1^) was added to a final concentration of 30 g l^−1^. After 4 days’ dark incubation at 20 °C, explants were washed in a solution of filter-sterilised ticarcillin disodium/clavulanate potassium (TCA, Duchefa T0190) (400 mg l^−1^) in water, blotted and transferred to SRM selection medium containing filter-sterilised TCA (400 mg l^−1^) and kanamycin sulphate monohydrate (Kanamycin, Duchefa K0126) (100 mg l^−1^). ‘Hawaii 4’ petioles were initially cultured in Sarstedt Cell Culture Flasks (T-25) containing 15 ml liquid selection medium for 4 weeks, 30–50 explants per flask, shaking at 60 rpm at low light intensity at 20 °C, before transfer to semi-solid selection medium. Leaves were cultured throughout on semi-solid selection medium at 20 °C: approximately 10 leaves (abaxial side in contact with the medium) or 20 petiole pieces were cultured per 90 mm triple-vent Petri dish containing approximately 30 ml medium. Dishes were sealed with Parafilm^®^ M and incubated in the growth room as for shoot cultures. Subculture intervals were 4–6 weeks. At the second culture passage, half of the explants were transferred to SRM supplemented with gibberellic acid (GA_3_) (1 mg l^−1^) before autoclaving. Petioles were divided to separate calli developing at both petiole ends. Leaves were divided as expansion occurred to ensure contact with the selection medium, to facilitate sub culturing and to separate regenerating calli. Calli with shoots were harvested over a 7-month period, and transferred to rooting medium: MS medium 2.2 g l^−1^, supplemented with sucrose 20 g l^−1^, BAP 0.1 mg l^−1^, IBA 0.1 mg l^−1^, solidified with 9 g l^−1^ Daishin agar, pH adjusted to 5.8 before autoclaving. Filter-sterilised kanamycin (50 mg l^−1^) and TCA (400 mg l^−1^) were added after autoclaving. For shoots with an obvious albino phenotype, kanamycin was omitted from the medium. Albino and variegated shoots were sub cultured onto rooting medium with TCA (400 mg l^−1^). Control (WT) shoots were regenerated using the same method, except that the explants were not co-cultivated with *Agrobacterium* and antibiotics were omitted from the culture media.

### Analysis of transgenic lines

DNA was extracted from leaf material using the DNeasy Plant Kit (Qiagen, Manchester, UK). Primers used in PCR and sequencing reactions (Additional file 12: Table S5, Fig. 1) were synthesised by IDT Ltd. PCR screening of putative transgenic lines for Nos-Kan cassette and sgRNA insertions was performed using PCRBIO Taq Mix Red (PCR Biosystems) and reaction conditions as detailed in Additional file 13: Table S6. PCR amplicons spanning the target region or Left Border T-DNA-genomic DNA junction sites were sequenced by Illumina MiSeq: primers incorporated target-specific sequences at the 3’ ends, and Illumina adapter overhang sequences (P5 forward overhang, P7 reverse overhang) at the 5’ ends. A 401 bp genomic region was amplified using primer pair P5-300 F and P7-700F, binding 186 -> 161 bp upstream and 195 -> 170 downstream of the target region, respectively. Amplicons were prepared for sequencing following Illumina guide 16S Metagenomic Sequencing Library Preparation (Part # 15044223 Rev. B). Template was amplified using Q5^®^ Hot Start High-Fidelity 2X Master Mix (M0494, New England Biolabs Ltd.) and purified using Mag-Bind^®^ TotalPure NGS (M1378, Omega Bio-tek Inc.). Dual indices and Illumina sequencing adapters were attached using Nextera XT Index Kit (15055290, Illumina) and 2x KAPA HiFi HotStart ReadyMix (KK2605, Kapa Biosystems). PhiX control V3 (15017660, Illumina) and the amplicon library were diluted to 4 pM concentration and the sample was spiked with 30% PhiX before loading onto a MiSeq Reagent Nano Kit v2 500 cartridge (MS-103-1003, Illumina).

TAIL PCR was used to amplify T-DNA-genomic DNA junctions using PCRBIO Taq Mix Red (PCR Biosystems Ltd): first round PCR was performed using arbitrary degenerate primer AD3 [57] and T-DNA-specific primer TAIL R1 (binding 257 -> 228 downstream of the Left Border). Nested primer TAIL R2 (binding 209 -> 188 downstream of the left border) and AD3 were used for the second round PCR. PCR reactions were performed using a Verity 96-Well Thermal Cycler (#4375786, maximum block ramp of 3.9 °C/Sec and a maximum sample ramp of 3.35 °C/Sec). The PCR cycle was essentially as Liu et al. [57] with minor modifications (Additional file 13: Table S6). Amplicons were purified using Mag-Bind^®^ TotalPure NGS and Sanger-sequenced by Eurofins Genetic Services Ltd. The sequencing primer (TAIL SEQ) binds 177 -> 146 downstream of the T-DNA left border.

### Sequencing analysis and software

Illumina sequencing reads were trimmed to remove low quality data and Illumina adapters with fastq-mcf (http://code.google.com/p/ea-utils) before being aligned to the reference *F. vesca* ‘Hawaii 4’ genome v4.0.a1 [9], using BWA v0.7.15 [58]. Variants in respect to the reference genome were predicted and quantified from aligned reads using the CrispRVariant package v1.9.2 [59]. Nucleotide and protein alignments were performed using Geneious version 10.0.2 (http://www.geneious.com) [60]. Schematic maps were prepared using IBS software [61].

## Additional files

**Additional file 1: Table S1.** Sequences of guide RNA constructs.

**Additional file 2: Fig. S1.** Sequence mutations and corresponding phenotypes of CRISPR/Cas9 transgenic lines of ‘Hawaii 4’.

**Additional file 3: Fig. S2.** Sequence mutations and corresponding phenotypes of CRISPR/Cas9 transgenic lines of ‘Calypso’.

**Additional file 4: Table S2.** Summary of analyses of CRISPR/Cas and wild-type ‘Calypso’ and ‘Hawaii 4’ shoot lines and samples. Sample entries for shoot lines/shoots/samples are pattern and colour coded: solid green, pale green or pale brown shading = green, pale green or off-white tissue, respectively; mottled shading = variegated tissue; no shading = white tissue. Results of PCR screening for presence of T-DNA Nos-Kan cassette and sgRNA insertion are indicated by ‘+’ (amplification of expected product) or ‘-’ (no product). Sequence results obtained for TAIL PCR reactions are indicated by ‘+’; ‘---’ = not done; F - failed sequence analysis. % wild type (WT) reads are shown for target site amplicon sequences: bold font for % WT reads and asterisks highlight results which may be unexpected in relation to observed phenotype. Mutant sequence variant type and putative allele status are shown for each sample.

**Additional file 5: Table S3.** Summary of ‘Calypso’ and ‘Hawaii 4’ samples: correlation of sequence data with phenotype.

**Additional file 6: Fig. S3.** Gel electrophoresis of target site amplicons.

**Additional file 7: Fig. S4.** Mutations of the *PDS* gene in CRISPR/Cas9 transgenic lines of ‘Hawaii 4’ and ‘Calypso’.

**Additional file 8: Fig. S5.** Gel electrophoresis of TAIL PCR amplification products for Calypso (a) and Hawaii 4 (b–d).

**Additional file 9: Fig. S6.** Left border T-DNA integration.

**Additional file 10: Table S4.** TAIL PCR amplicon sequences for transgenic shoot lines of ‘Hawaii 4’ and ‘Calypso’.

**Additional file 11: Fig. S7.** Amino acid sequences for published plant *PDS* genes aligned against *Fragaria vesca*.

**Additional file 12: Table S5.** Primer sequences.

**Additional file 13: Table S6.** PCR cycles used for analysis of plant samples.

## Abbreviations

CRISPR: clustered regularly interspaced short palindromic repeats
Cas9: CRISPR associated DNA-cutting enzyme
sgRNA: single-guide RNA
GA_3_: gibberellic acid
WT: wild type
TAIL PCR: thermal asymmetric interlaced PCR
PCR: polymerase chain reaction
T-DNA: transfer DNA
GFP: green fluorescent protein
BLAST: basic local alignment search tool
PAM: protospacer-adjacent motif
NGS: next generation sequencing
