# Supplementary material for: CRISPR/Cas9-mediated mutagenesis of phytoene desaturase in diploid and octoploid strawberry
Source: Plant Methods. 2019 May 2;15:45. doi: 10.1186/s13007-019-0428-6 (PMC6495592; doi:10.1186/s13007-019-0428-6)
Supplement: Supplementary file 1 — Additional file 1: Table S1. Sequences of guide RNA constructs. [file 13007_2019_428_MOESM1_ESM.pdf]

Supplementary Information

(a)
GGAGTGATCAAAAGTCCCACATCGATCAGGTGATATATAGCAGCTTAGTTTATATAA
*TGATAGAGTCGACATAGCGATTG***ACCTGATCGAGTAACTACTG**gttttagagctagaaatag
caagttaaaataaggctagtccgttatcaacttgaaaaagtggcaccgagtcggtgc*TTTTTTTTGCAAAATTTT*
CCAG
(b)
AGGCTACGAAATAATCCCACATCGGAAACCTCTGTCTACAAGGACTTCTTTATATAC
*AATTGACTCCCATCTAAGCTTG***ACCTGATCGAGTAACTACTG**gttttagagctagaaatagca
agttaaaataaggctagtccgttatcaacttgaaaaagtggcaccgagtcggtgc*TTTTTTTGCAATTTTTTGC*
AA

Table S1. Sequences of guide RNA constructs.
(a) *AtPDS*74 incorporating *Arabidopsis U6-26* promoter consensus sequence and terminator sequence; (b) *FvPDS*74 incorporating *F. vesca U6III* promoter sequence and terminator sequence, FvAssembly 1.1, LG4. Components are denoted as follows: promoter and terminator sequences are italicised; the target guide sequence ‘Fv74’ is in bold font; the scaffold sequence is in lower case font; an additional guanine base preceding the guide sequence is underlined.
