# Supplementary material for: CRISPR/Cas9-mediated mutagenesis of phytoene desaturase in diploid and octoploid strawberry
Source: Plant Methods. 2019 May 2;15:45. doi: 10.1186/s13007-019-0428-6 (PMC6495592; doi:10.1186/s13007-019-0428-6)
Supplement: Supplementary file 2 — Additional file 2: Fig. S1. Sequence mutations and corresponding phenotypes of CRISPR/Cas9 transgenic lines of ‘Hawaii 4’. [file 13007_2019_428_MOESM2_ESM.pdf]

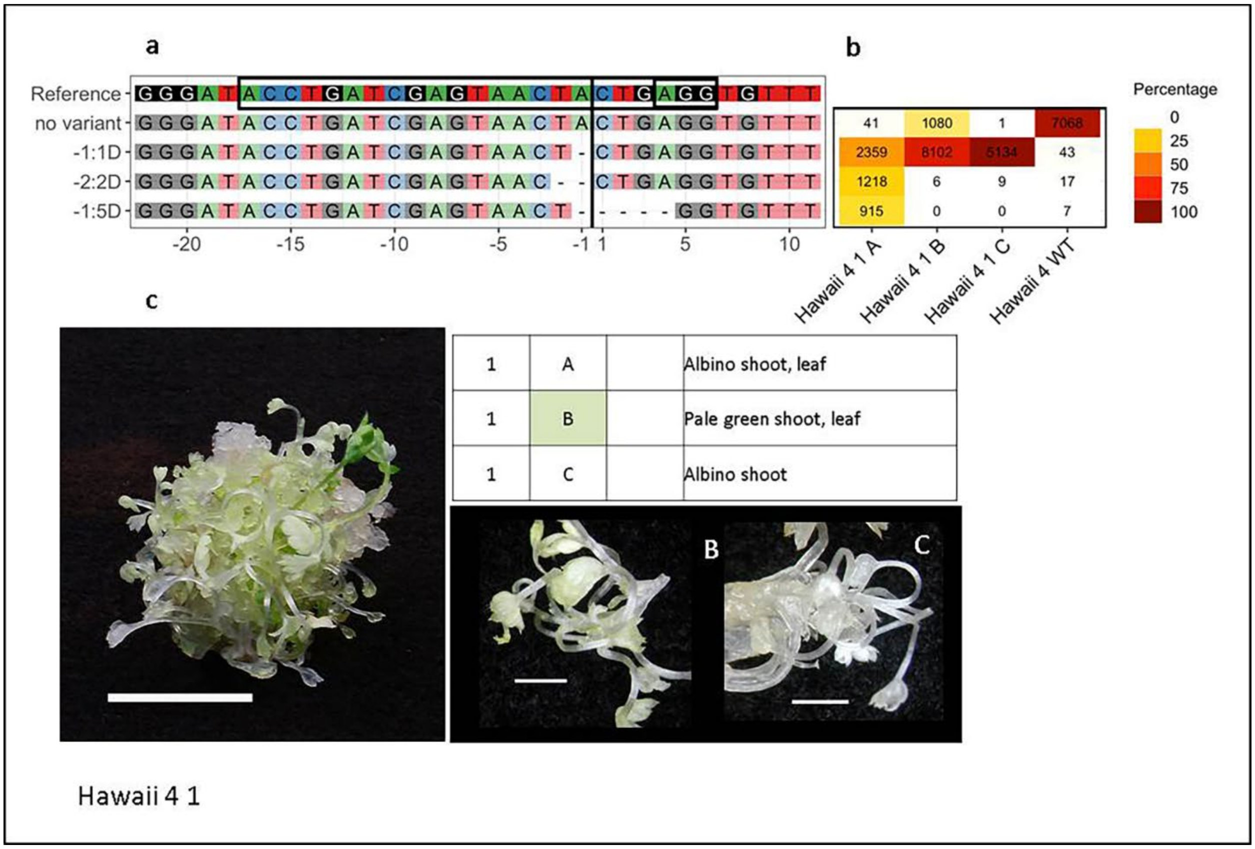
a
Reference GGGATACCTGATCGAGTAACTACTGAGGTGTTT
no variant GGGATACCTGATCGAGTAACTACTGAGGTGTTT
-1:1D GGGATACCTGATCGAGTAACT-CTGAGGTGTTT
-2:2D GGGATACCTGATCGAGTAAC--CTGAGGTGTTT
-1:5D GGGATACCTGATCGAGTAACT-----GGTGTTT
-20 -15 -10 -5 -1 1 5 10
b
Percentage
0
25
50
75
100
41 1080 1 7068
2359 8102 5134 43
1218 6 9 17
915 0 0 7
Hawaii 4 1 A
Hawaii 4 1 B
Hawaii 4 1 C
Hawaii 4 WT
c
1 A Albino shoot, leaf
1 B Pale green shoot, leaf
1 C Albino shoot
B
C
Hawaii 4 1

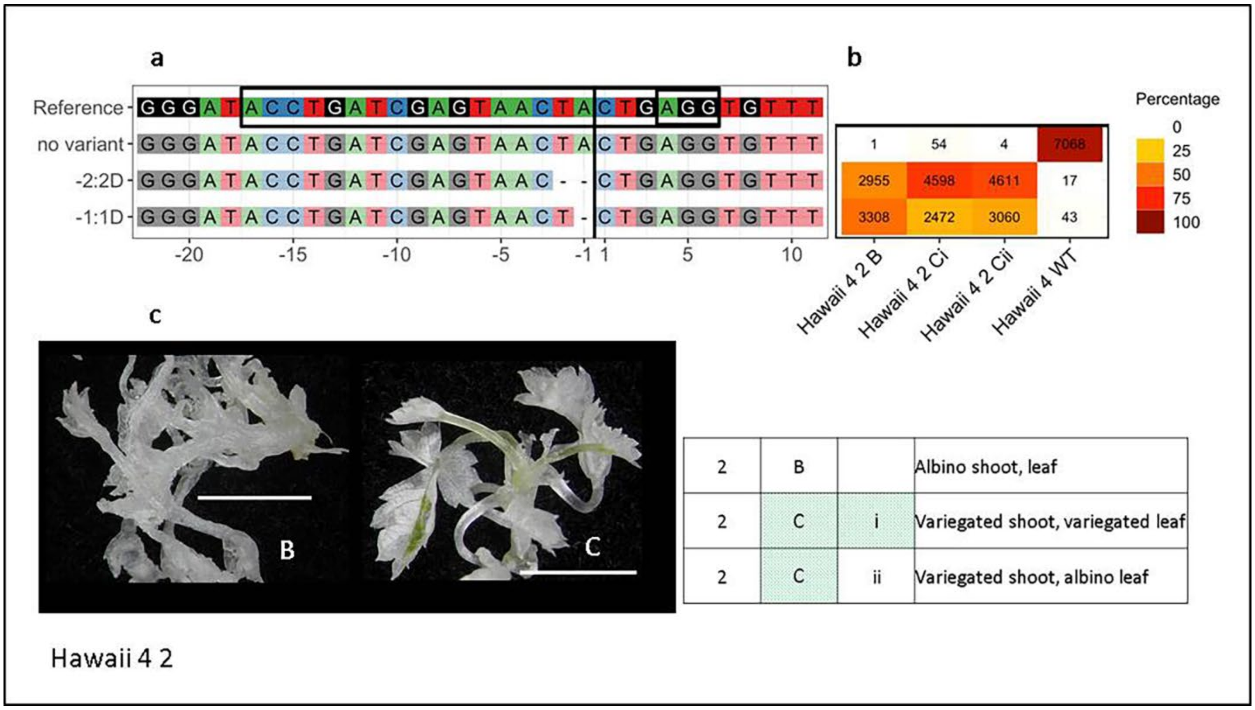

2	B		Albino shoot, leaf
2	C	i	Variegated shoot, variegated leaf
2	C	ii	Variegated shoot, albino leaf

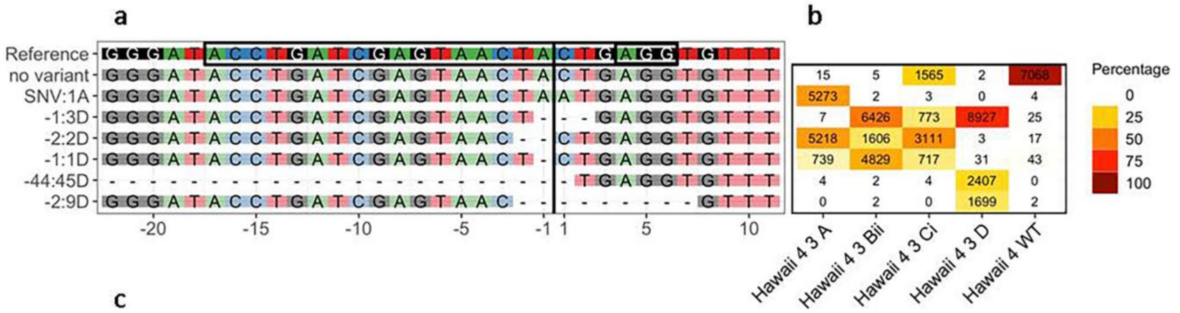

c

3	A		Green shoot rooted on kanamycin	3	C	i	Variegated shoot, variegated leaf
3	B	ii	Albino axillary, leaf	3	D		Albino shoot

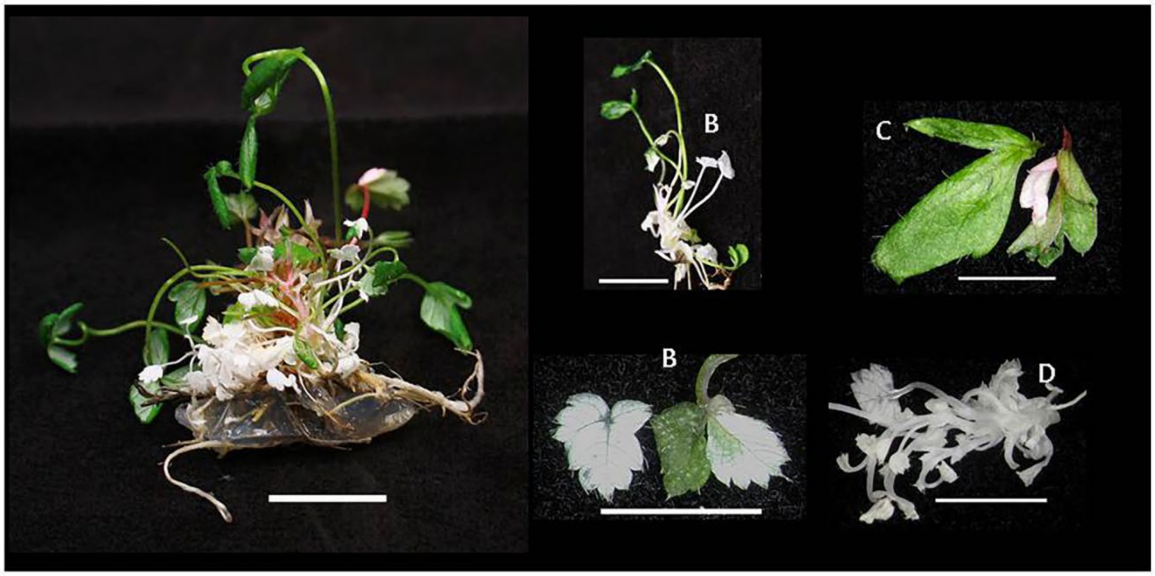

Hawaii 4 3

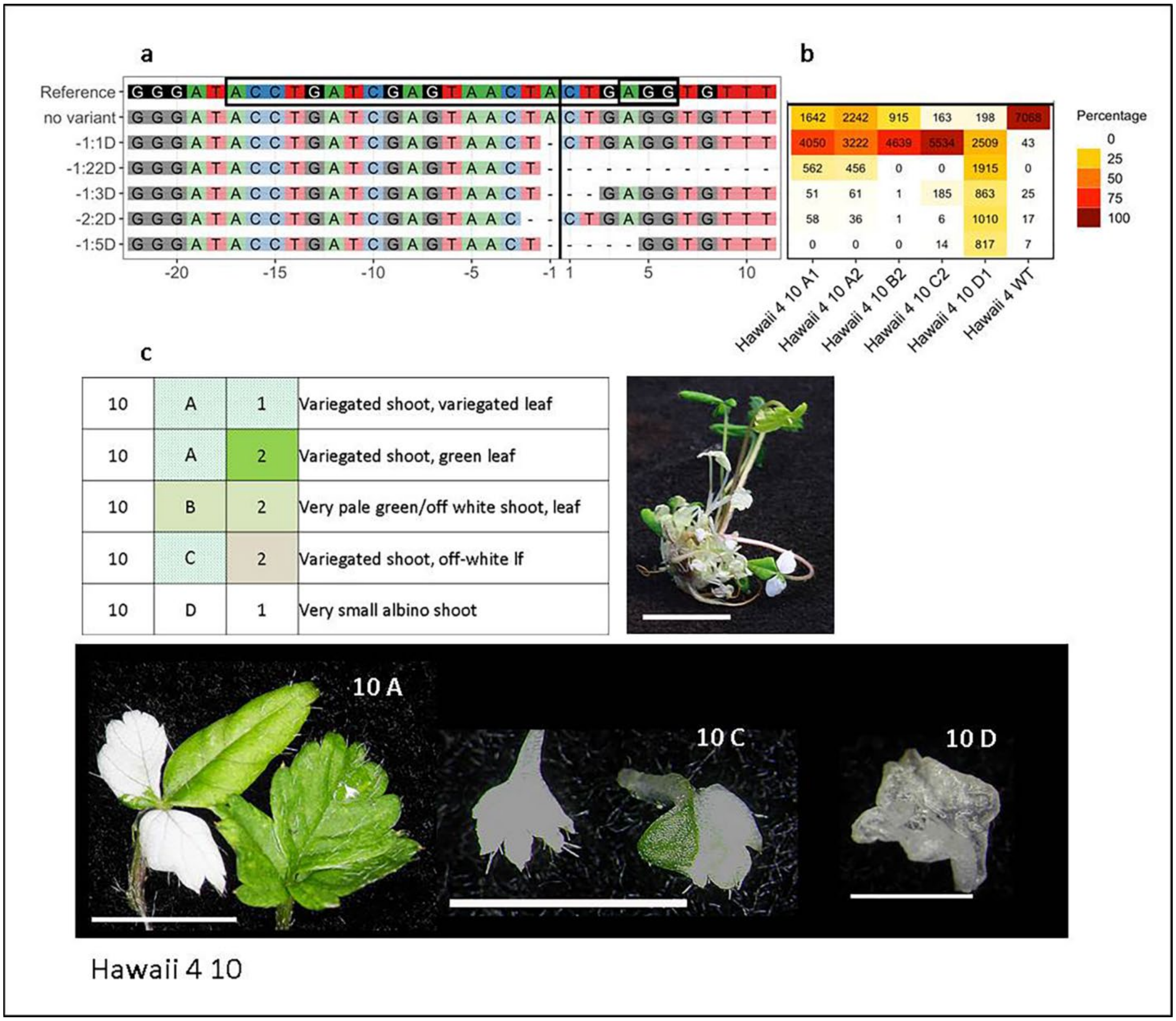
a
Reference
no variant
-1:1D
-1:22D
-1:3D
-2:2D
-1:5D
-20
-15
-10
-5
-1
1
5
10
b
1642 2242 915 163 198 7068
4050 3222 4639 5534 2509 43
562 456 0 0 1915 0
51 61 1 185 863 25
58 36 1 6 1010 17
0 0 0 14 817 7
Hawaii 4 10 A1
Hawaii 4 10 A2
Hawaii 4 10 B2
Hawaii 4 10 C2
Hawaii 4 10 D1
Hawaii 4 WT
Percentage
0
25
50
75
100
c
10 A 1 Variegated shoot, variegated leaf
10 A 2 Variegated shoot, green leaf
10 B 2 Very pale green/off white shoot, leaf
10 C 2 Variegated shoot, off-white lf
10 D 1 Very small albino shoot
10 A
10 C
10 D
Hawaii 4 10

a

b

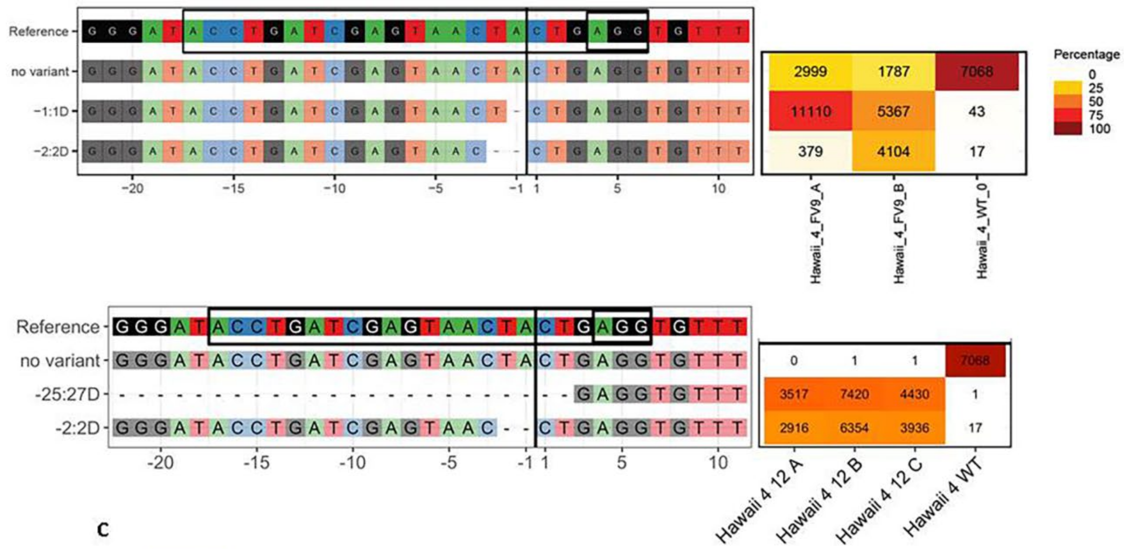

c

9 (Fv9)	A		Pale green shoot, leaf, mostly albino shoot cluster
9 (Fv9)	B		Albino shoot, leaf, mostly albino shoot cluster
12	A		Albino shoot, leaf
12	B		Albino shoot, leaf
12	C		Albino shoot, leaf

Hawaii 4 9, 12

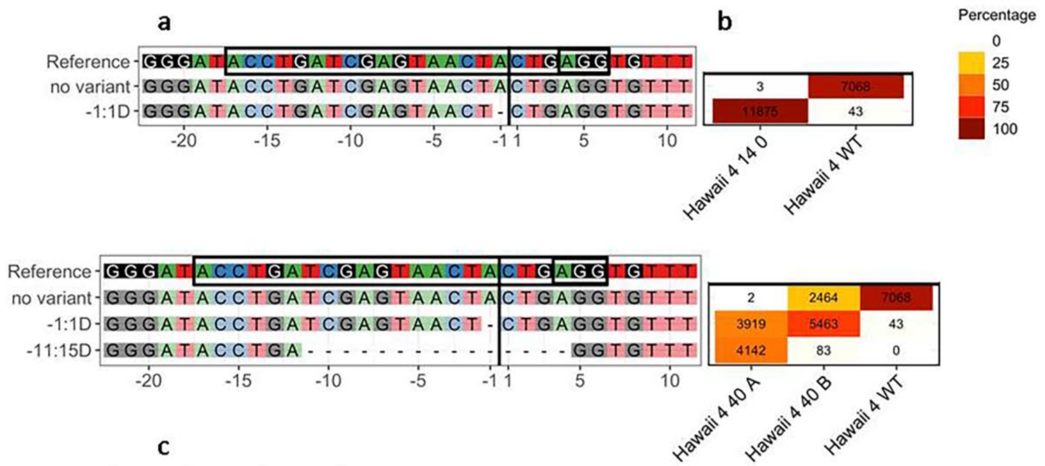

c

14			Albino shoot, leaf
40	A		Albino shoot
40	B		Variegated (mottled), separate rooted shoot

Hawaii 4 14, 40

a **b**

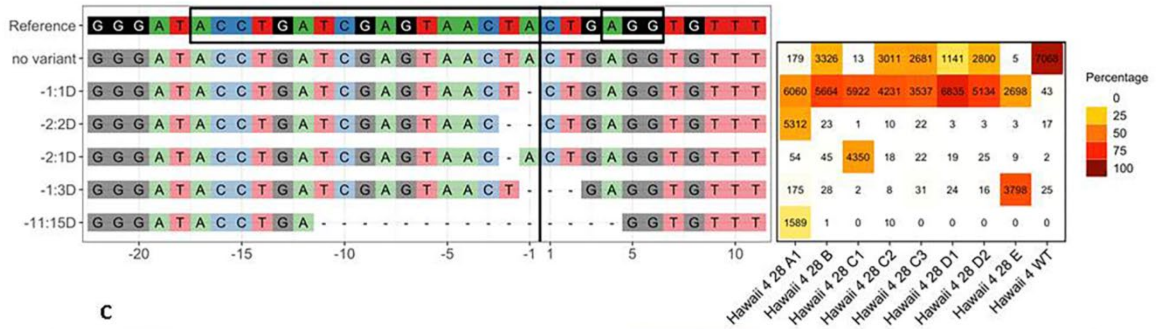

c

28	A	1	White axillary, variegated shoot	28	C	3	Green axillary shoot, green leaf
28	B		Variegated leaf, very small white sector	28	D	1	Variegated shoot, variegated leaf (very small green sector leaf edge tip)
28	C	1	Shoot axillary which is half green, half white, albino leaf	28	D	2	Variegated shoot, variegated leaf ('harlequin')
28	C	2	Shoot axillary which is half green, half white, green leaf	28	E		All-albino shoot cluster, leaf

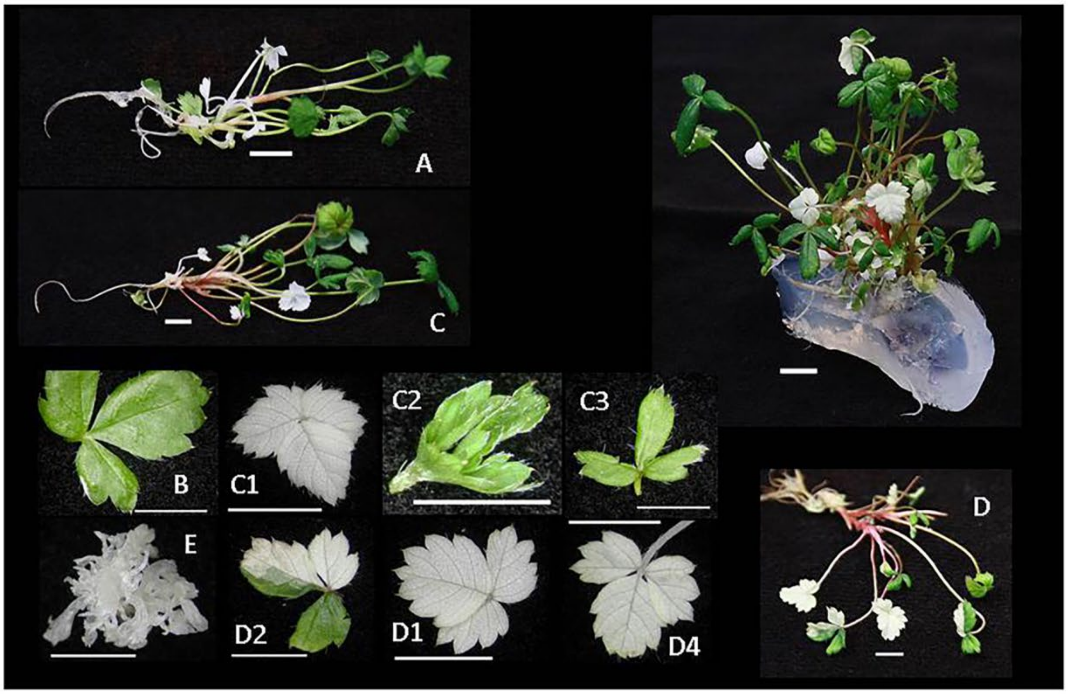

Hawaii 4 28

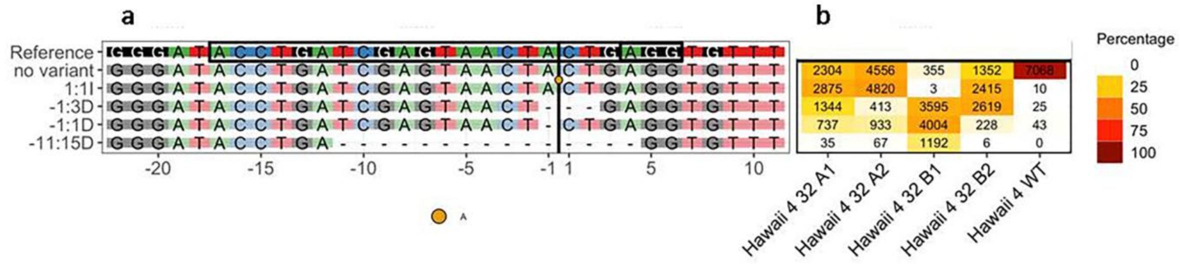

c

32	A	1	Variegated leaf, DNA from green middle leaflet in photo
32	A	2	Green leaf from variegated shoot
32	B	1	Variegated leaves at top half of shoot, off-white shoot
32	B	2	White leaf from base, off-white shoot

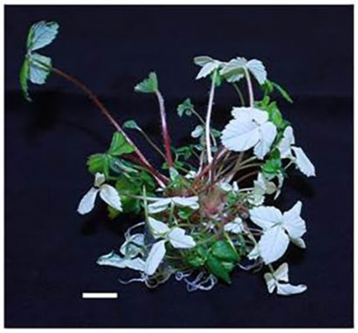

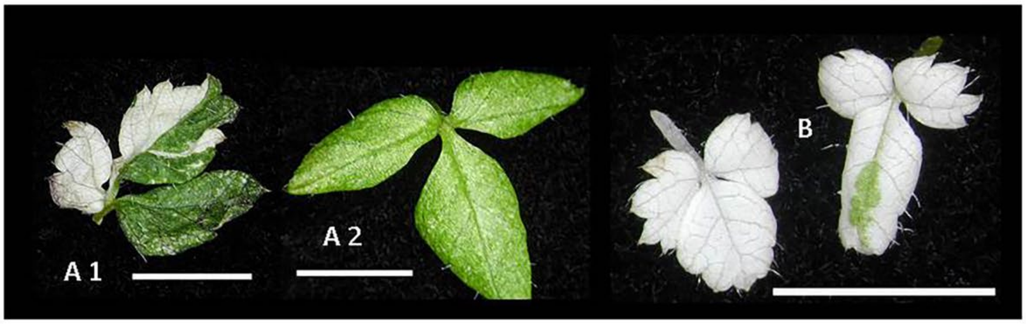

Hawaii 4 32

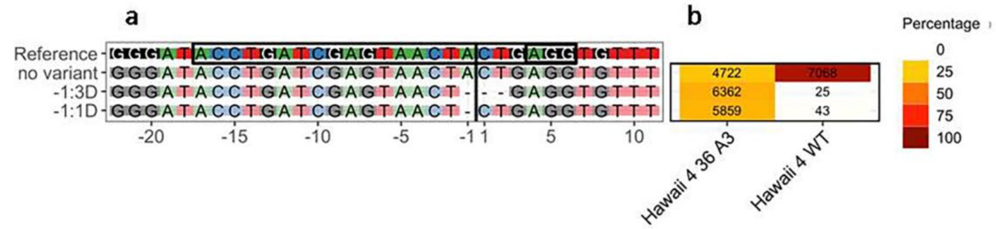

c

36	A	3	Variegated shoot, apical leaf - 2 leaflets with green fleck, 1 all white

Hawaii 4 36

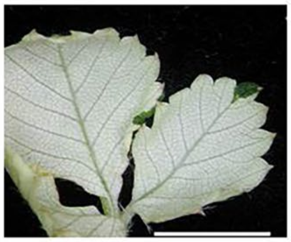

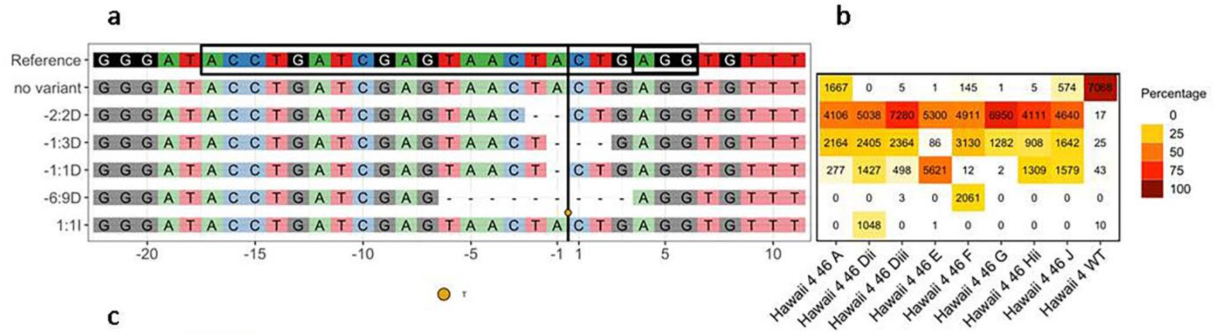

c

46	A		Green rooted shoot
46	D	ii	Albino leaf, from shoot with white and green leaves
46	D	iii	Apical albino leaves, green bits at base. Separate shoot from Dii
46	E		Small albino shoot
46	F		Small albino shoot
46	G		Rooted very pale shoot
46	H	ii	Variegated shoot, white basal leaf
46	J		Pale green shoot, leaf

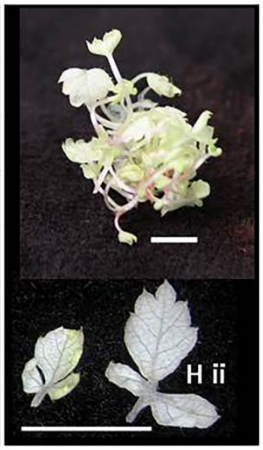

Hawaii 4 46

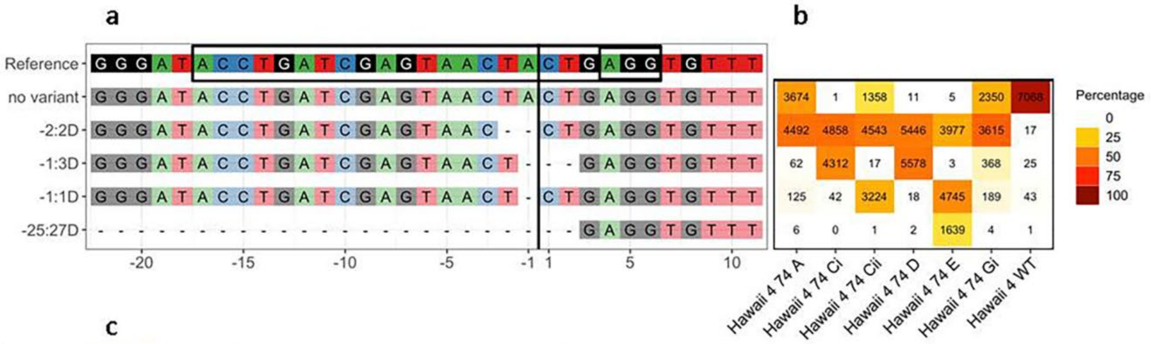

c

74	A		Green rooted shoot, leaf
74	C	i	Variegated shoot, albino axillary shoot, leaf
74	C	ii	Pale green axillary shoot, leaf
74	D		Rooted albino shoot
74	E		Albino shoot
74	G	i	Variegated shoot, variegated leaf

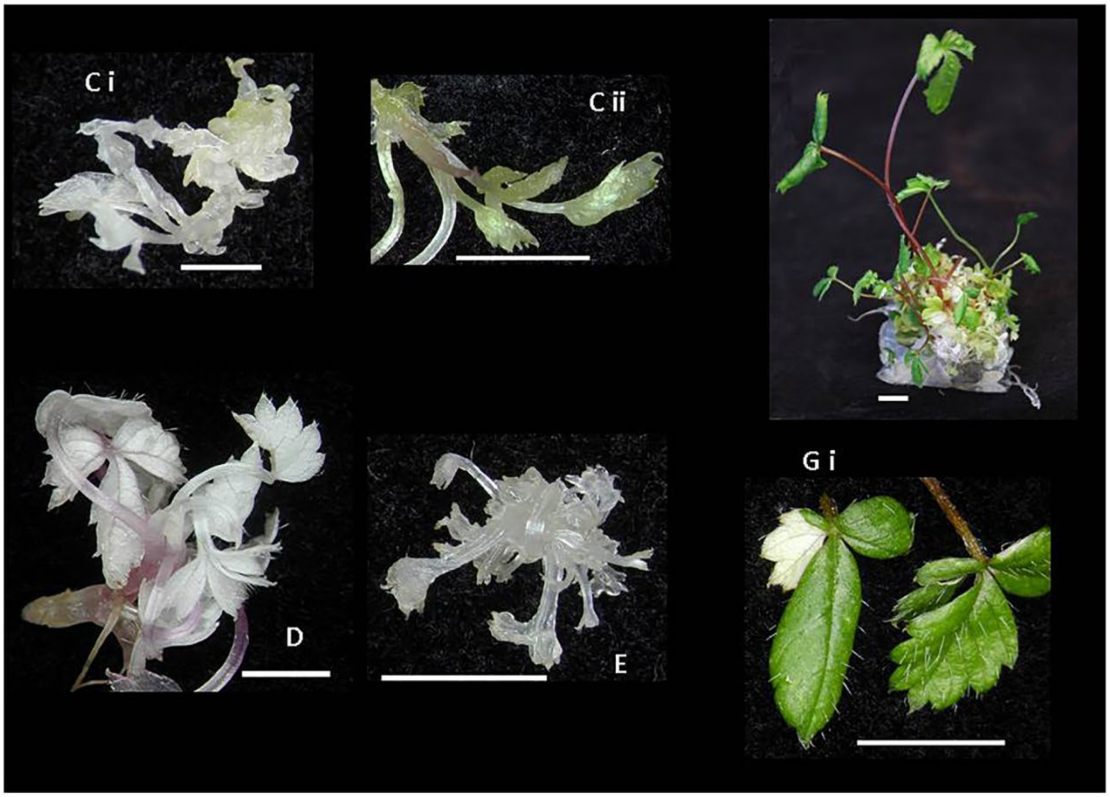

Hawaii 4 74

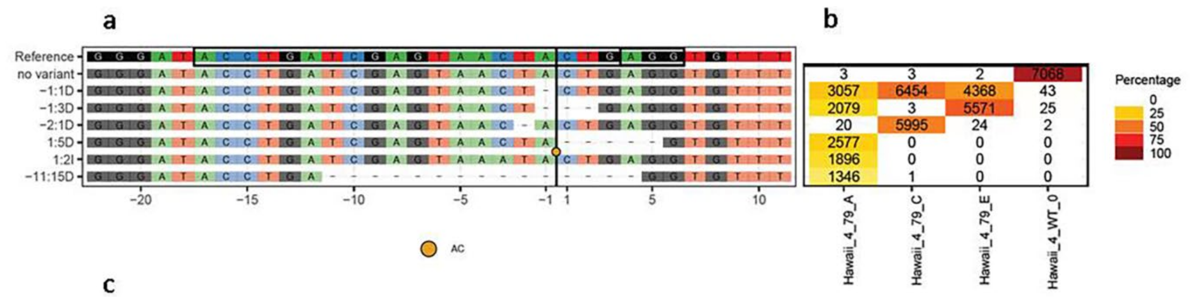

c

79	A		Albino shoot, leaf
79	C		Albino shoot, leaf
79	E		Albino shoot, leaf

Hawaii 4 79

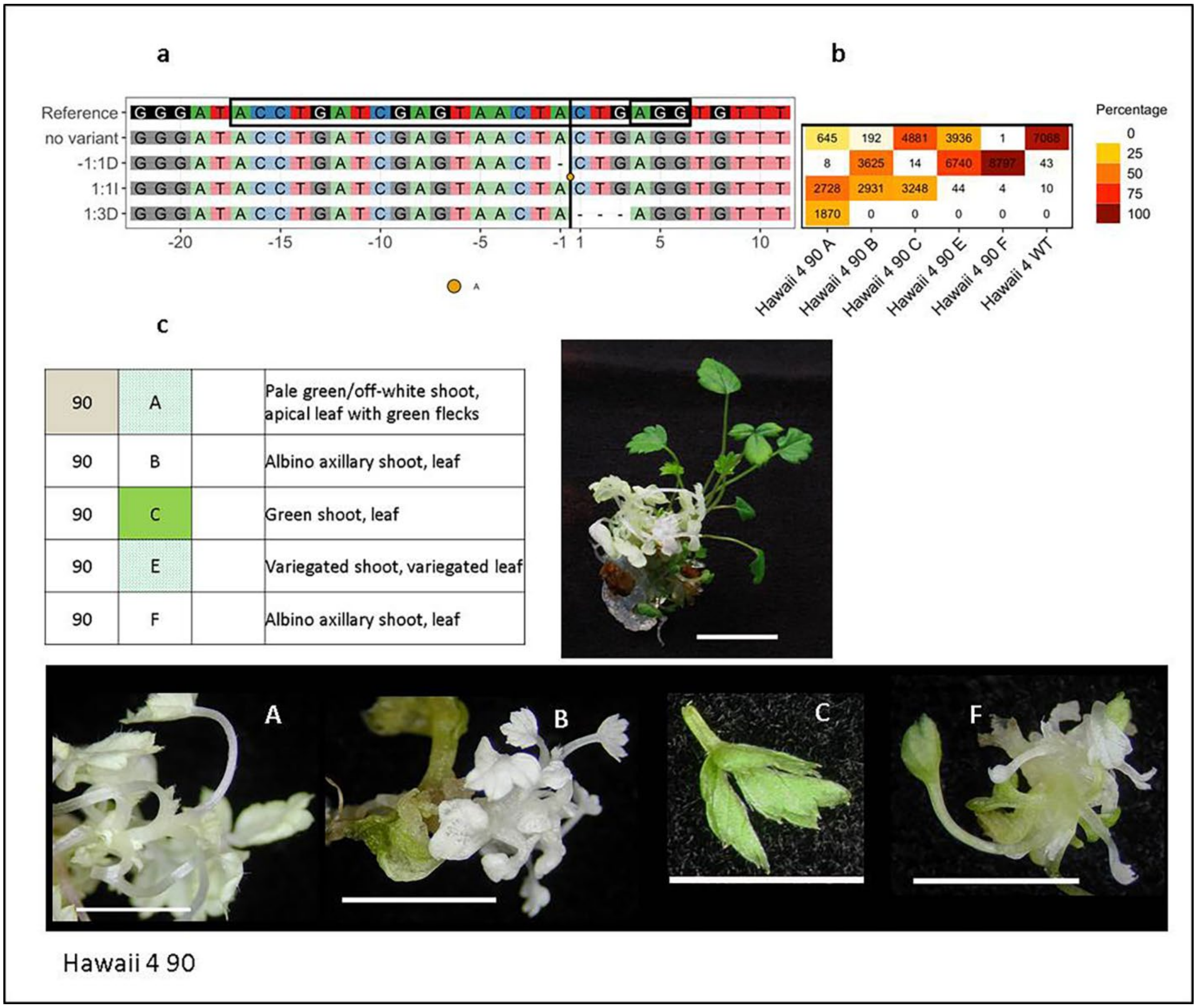
a
b
Reference
no variant
-1:1D
1:1I
1:3D
-20
-15
-10
-5
-1
1
5
10
A
Percentage
0
25
50
75
100
645 192 4881 3936 1 7068
8 3625 14 6740 8797 43
2728 2931 3248 44 4 10
1870 0 0 0 0 0
Hawaii 4 90 A
Hawaii 4 90 B
Hawaii 4 90 C
Hawaii 4 90 E
Hawaii 4 90 F
Hawaii 4 WT
c
90 A Pale green/off-white shoot, apical leaf with green flecks
90 B Albino axillary shoot, leaf
90 C Green shoot, leaf
90 E Variegated shoot, variegated leaf
90 F Albino axillary shoot, leaf
A
B
C
F
Hawaii 4 90

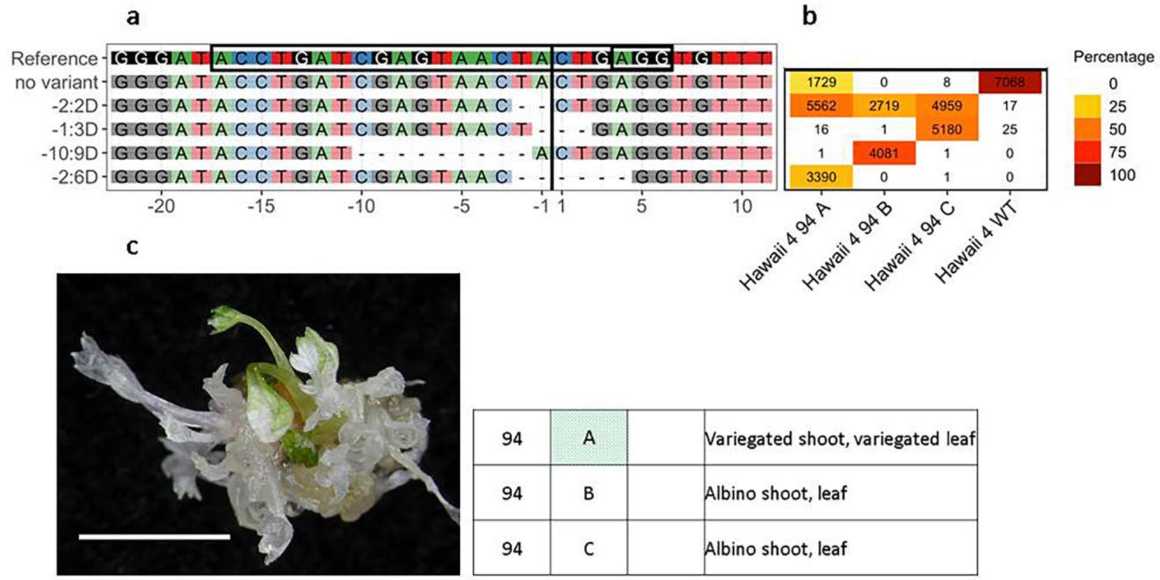

94	A		Variegated shoot, variegated leaf
94	B		Albino shoot, leaf
94	C		Albino shoot, leaf

Hawaii 4 94

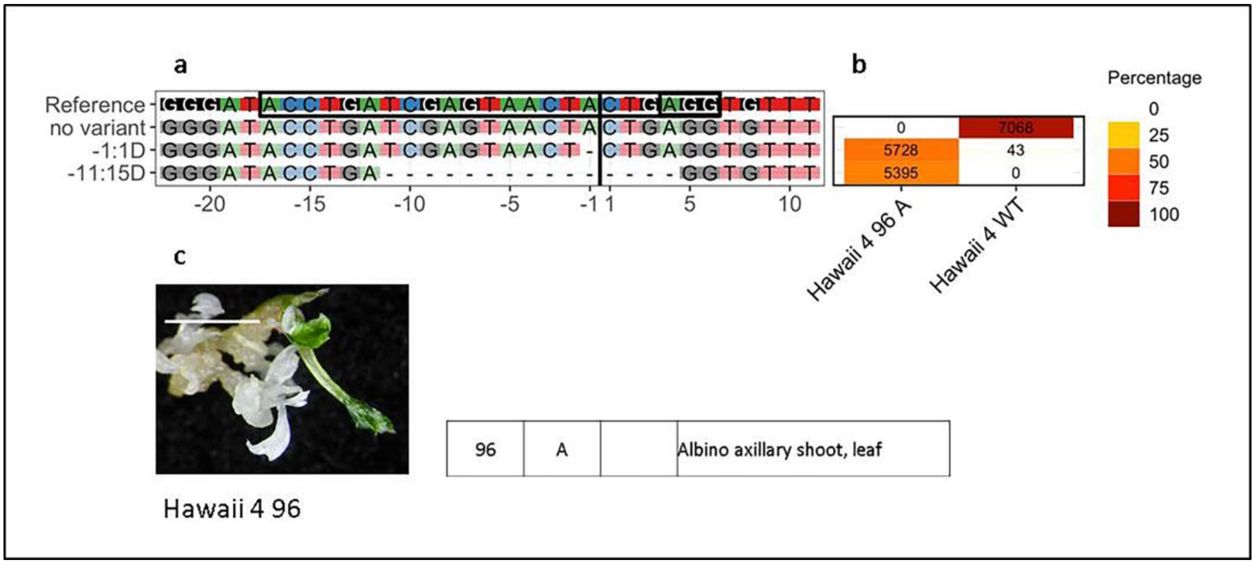

Fig. S1. Sequence mutations and corresponding phenotypes of CRISPR/Cas9 transgenic lines of 'Hawaii 4'.

Sequence mutations and allelic variants with corresponding phenotypes in transgenic shoot lines of *Fragaria* 'Hawaii 4'. **a** Alignment of the sequence for each variant to the reference sequence. To the left of the panel, each sequence variant type is identified by the location of the mutation relative to the cut site:number bases deleted (D), inserted (I), or substituted (SNV:location relative to cut site and base substitution). Insertion positions are indicated by an orange circle with the inserted base/s shown. Deletions of 3 bases or multiples of 3 result in amino acid loss. Other deletions and insertions cause frameshift and generate stop codons within exon 7, resulting in truncated PDS protein. Insertions and substitutions result in amino acid substitutions. The cut site is shown by a black vertical line; the target site and PAM site (AGG) are within the box in the reference sequence. **b** Heat map showing the number and percentage of variant reads for each sample. Samples are arranged in columns and identified below the panel. **c** Images of phenotypes and an accompanying table giving descriptions for each sequenced sample. Numbers in the first column refer to the shoot line. In subsequent columns individual shoots from a shoot line are designated using a letter (A, B, etc.) and numbers indicate individual leaves from individual shoots. White, pale green or dark green box shading indicates albino, pale green/variegated or green phenotype respectively, for shoots and leaves. WT=wild type. Scale bars are 5 mm.
