# Supplementary material for: CRISPR/Cas9-mediated mutagenesis of phytoene desaturase in diploid and octoploid strawberry
Source: Plant Methods. 2019 May 2;15:45. doi: 10.1186/s13007-019-0428-6 (PMC6495592; doi:10.1186/s13007-019-0428-6)
Supplement: Supplementary file 3 — Additional file 3: Fig. S2. Sequence mutations and corresponding phenotypes of CRISPR/Cas9 transgenic lines of ‘Calypso’. [file 13007_2019_428_MOESM3_ESM.pdf]

a **b**

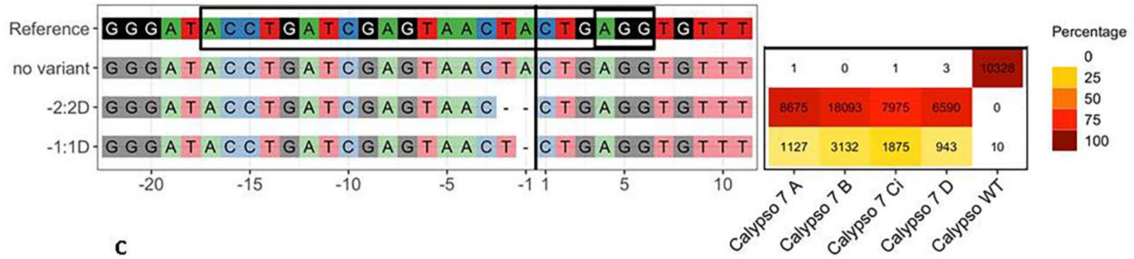

c

7	A		Albino shoot, leaf	7	Ci		Albino shoot, leaf
7	B		Albino shoot, leaf	7	D		Variegated shoot, variegated leaf?

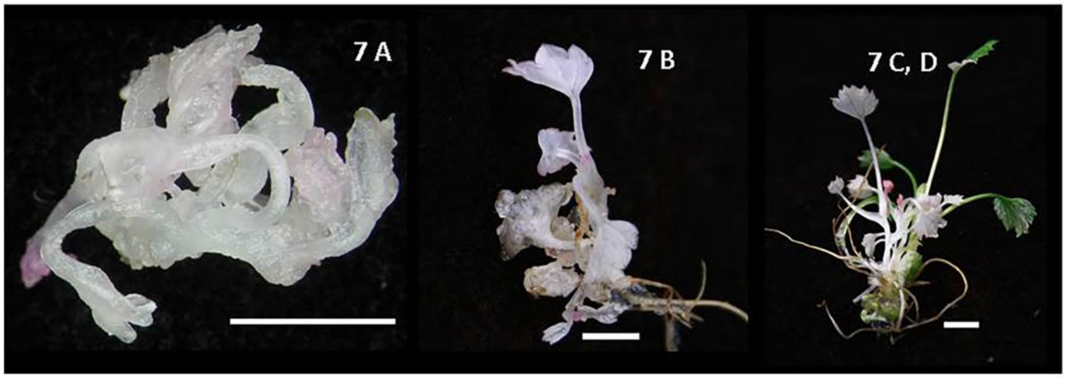

Calypso 7

a **b**

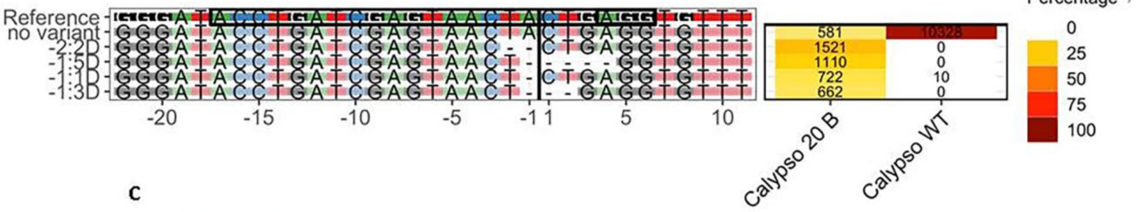

c

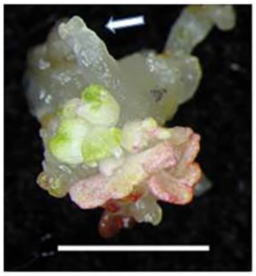

20	B		Mixed variegated and albino shoot line. Albino leaf from very small shoot.

Calypso 20 B

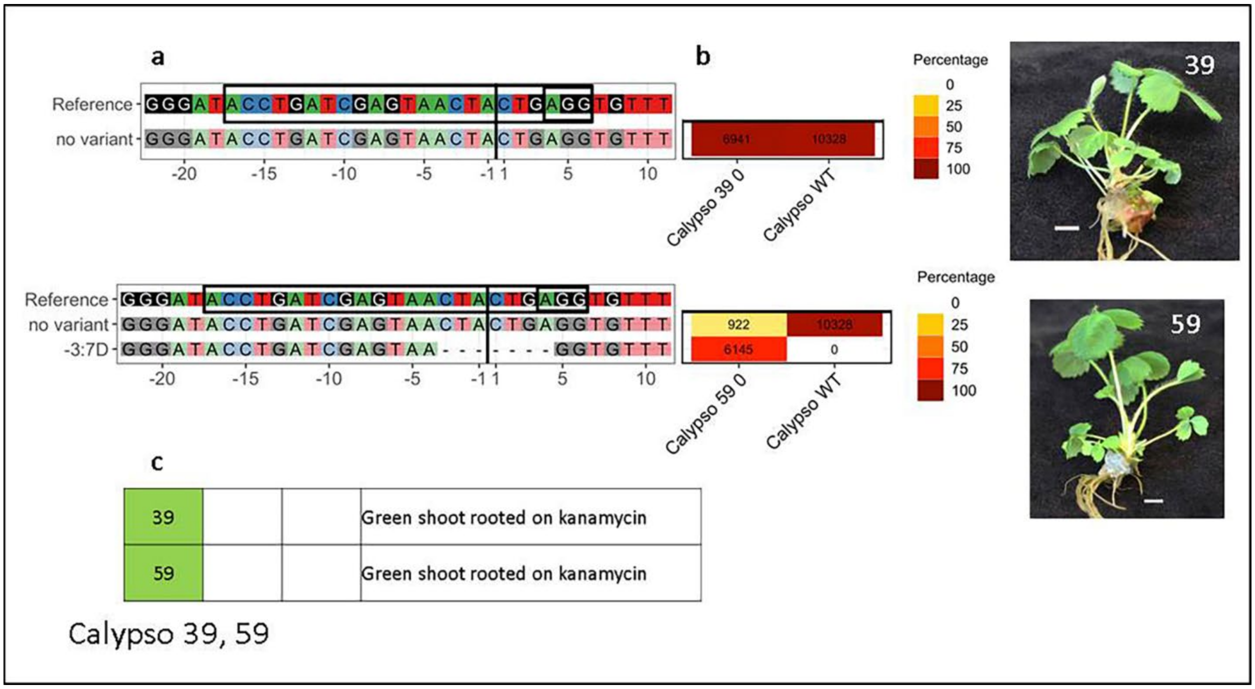
a
Reference
no variant
-20
-15
-10
-5
-1 1
5
10
b
Percentage
0
25
50
75
100
6941
10328
Calypso 39 0
Calypso WT
39
Reference
no variant
-3:7D
922
10328
6145
0
Calypso 59 0
Calypso WT
59
c
39
Green shoot rooted on kanamycin
59
Green shoot rooted on kanamycin
Calypso 39, 59

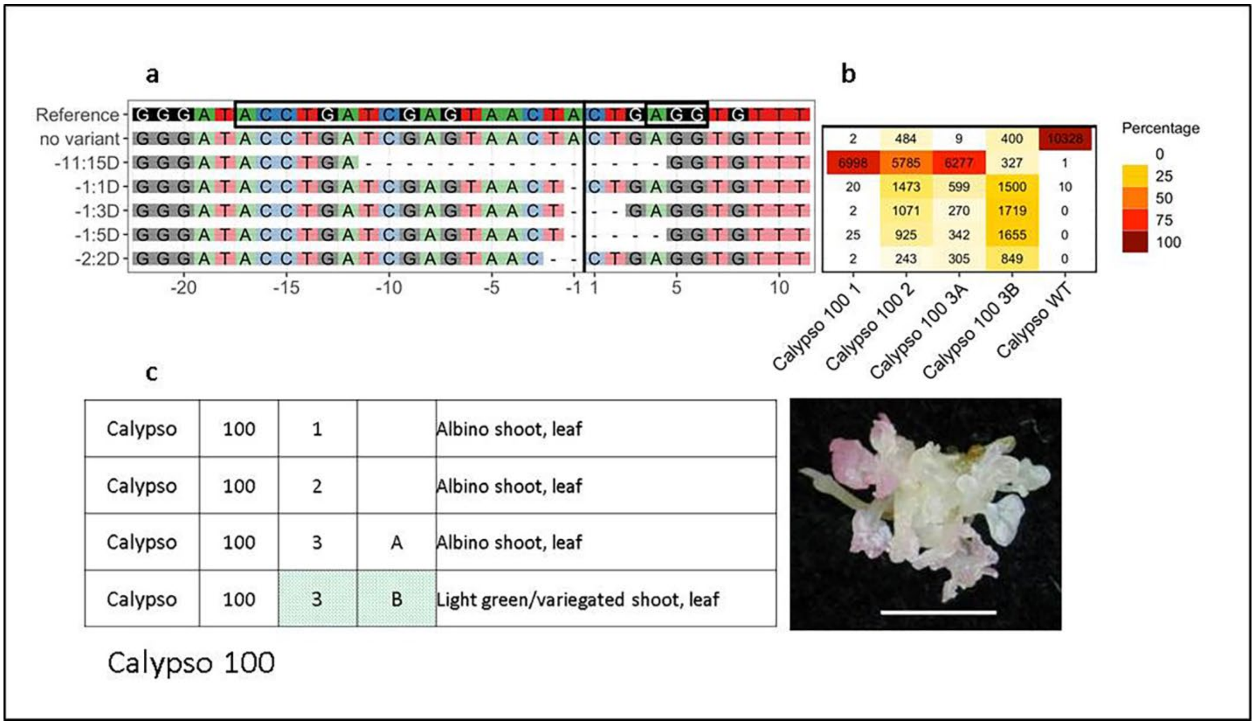

Fig. S2. Sequence mutations and corresponding phenotypes of CRISPR/Cas9 transgenic lines of 'Calypso'.

Sequence mutations and allelic variants with corresponding phenotypes in transgenic shoot lines of *Fragaria* 'Calypso'. **a** Alignment of the sequence for each variant to the reference sequence. To the left of the panel, each sequence variant type is identified by the location of the mutation relative to the cut site:number bases deleted (D). Deletions of 3 bases or multiples of 3 result in amino acid loss. Other deletions cause frameshift. All deletions generate stop codons within exon 7, resulting in truncated PDS protein. The cut site is shown by a black vertical line; the target site and PAM site (AGG) are within the box in the reference sequence. **b** Heat map showing the number and percentage of variant reads for each sample. Samples are arranged in columns and identified below the panel. **c** Images of phenotypes and an accompanying table giving descriptions for each sequenced sample. Numbers in the first column refer to the shoot line. In subsequent columns individual shoots from a shoot line are designated using a letter (A, B, etc.) and numbers indicate individual leaves from individual shoots. White, pale green or dark green box shading indicates albino, pale green/variegated or green phenotype respectively, for shoots and leaves. WT=wild type. Scale bars are 5 mm.
