# Supplementary material for: CRISPR/Cas9-mediated mutagenesis of phytoene desaturase in diploid and octoploid strawberry
Source: Plant Methods. 2019 May 2;15:45. doi: 10.1186/s13007-019-0428-6 (PMC6495592; doi:10.1186/s13007-019-0428-6)
Supplement: Supplementary file 5 — Additional file 5: Table S3. Summary of ‘Calypso’ and ‘Hawaii 4’ samples: correlation of sequence data with phenotype. [file 13007_2019_428_MOESM5_ESM.pdf]

Phenotype can be predicted from the proportion of WT and variant sequence reads				WT sequence reads are high in relation to phenotype observed				
Albino biallelic variants regenerating from callus	Albino shoot tip or axillary developing from variegated or pale green shoot	Pale green/off white tissue	Green/var (variant/WT) tissue	Albino tissue, higher than expected proportion of WT reads	Green or variegated tissue, single allele sequence variants	Tissue with possible chimaeric origin	Shoots showing evidence of continued gene editing	Cultivar
1A, C		1B						'Hawaii 4'
2B			2Ci				2C	
3Bii, D			3Ci					
			9A	9B				
		10B2, C2	10A1	10D1	10A2			
12								
14								
28C1,E			28D2	28A1	28B, C2, C3	28D1	28C	
			32B1		32A1, A2, B2	32B1, B2		
						36A3		
	39D		39A, C1, C2, C3, C4	39B			39C, D	
40A			40B					
46E, G, Hii	46Diii	46J		46F	46A		46D, H	
74D, E	74Ci	74Cii	74Gi		74A		74C	
79A, C, E								
89								
	90 F	90A	90E	90B	90C			
94B, C			94A					
	96A							
1								'Calypso'
5A, B			5D	5Ci			5C	
7A, B, C								
				20B				
					59			
				94				
100-1, 3A			100-3B	100-2				

Table S3. Summary of ‘Calypso’ and ‘Hawaii 4’ samples: correlation of sequence data with phenotype.
