# Supplementary material for: CRISPR/Cas9-mediated mutagenesis of phytoene desaturase in diploid and octoploid strawberry
Source: Plant Methods. 2019 May 2;15:45. doi: 10.1186/s13007-019-0428-6 (PMC6495592; doi:10.1186/s13007-019-0428-6)
Supplement: Supplementary file 6 — Additional file 6: Fig. S3. Gel electrophoresis of target site amplicons. [file 13007_2019_428_MOESM6_ESM.pdf]

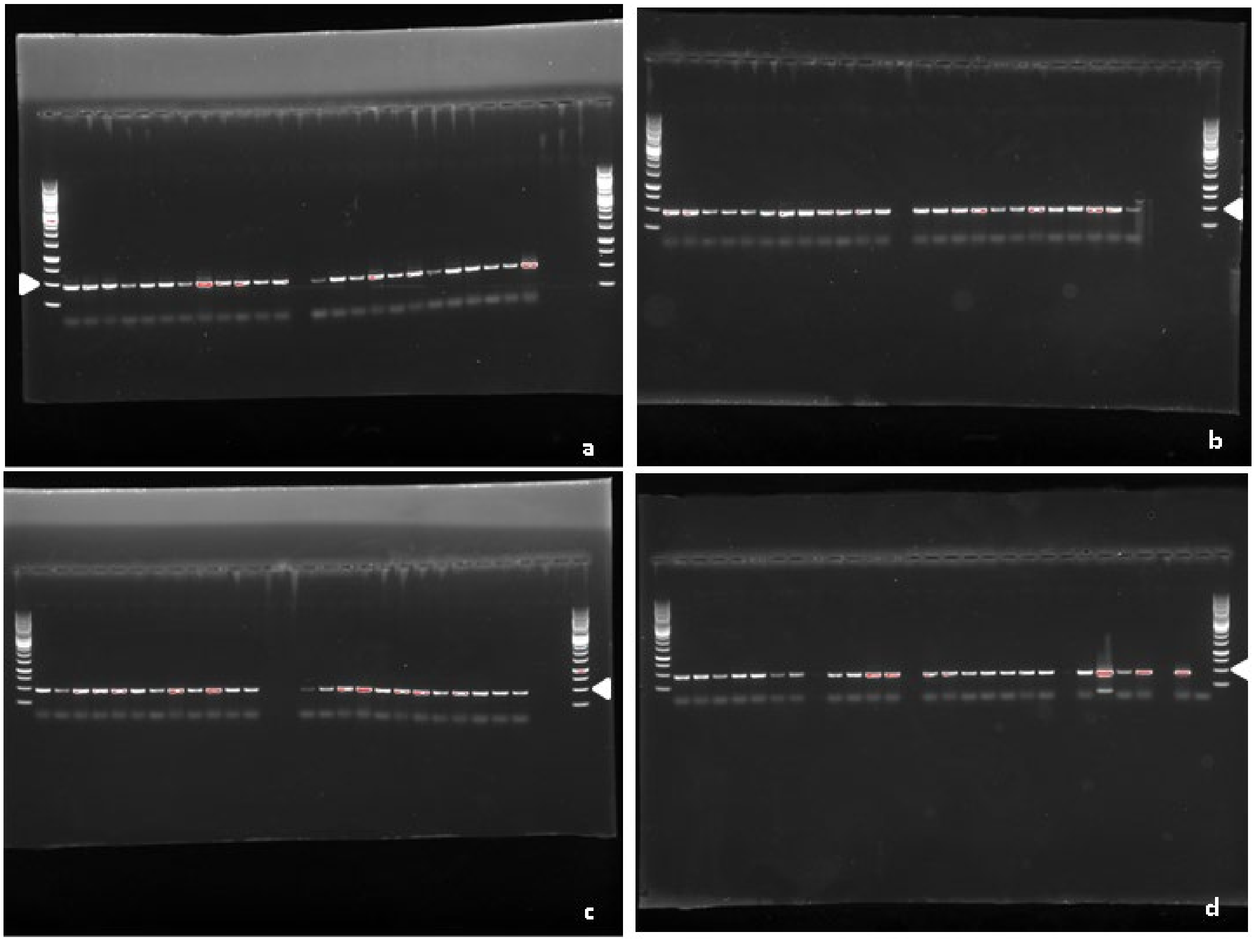

Fig. S3. Gel electrophoresis of target site amplicons.
A 401 bp genomic region was amplified using primer pair P5-300 F and P7-700F, binding 186 ->161 bp upstream and 195 -> 170 downstream of the target region, respectively: **a** ‘Calypso’ lines; **b** - **d** ‘Hawaii 4’ lines. The arrows indicate 500 bp ladder size marker.
