# Supplementary material for: CRISPR/Cas9-mediated mutagenesis of phytoene desaturase in diploid and octoploid strawberry
Source: Plant Methods. 2019 May 2;15:45. doi: 10.1186/s13007-019-0428-6 (PMC6495592; doi:10.1186/s13007-019-0428-6)
Supplement: Supplementary file 7 — Additional file 7: Fig. S4. Mutations of the PDS gene in CRISPR/Cas9 transgenic lines of ‘Hawaii 4’ and ‘Calypso’. [file 13007_2019_428_MOESM7_ESM.pdf]

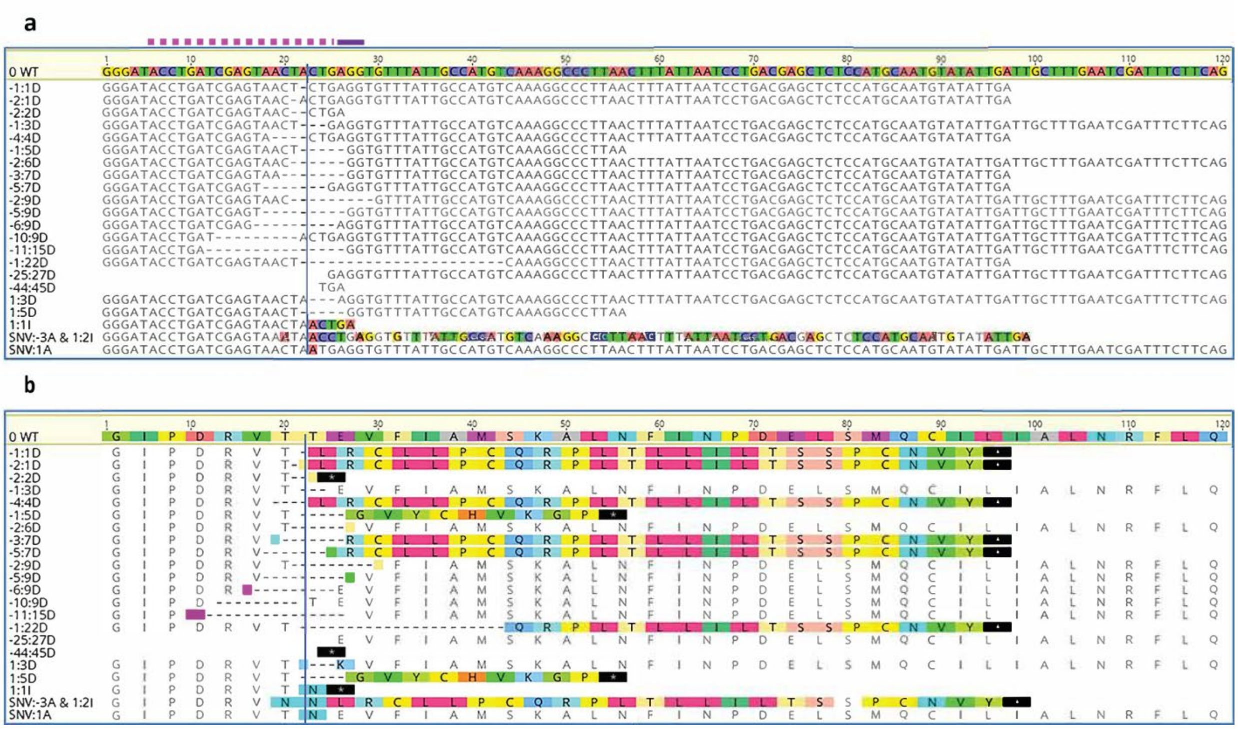

Fig. S4. Mutations of the *PDS* gene in CRISPR/Cas9 transgenic lines of ‘Hawaii 4’ and ‘Calypso’.

a Summary of CRIPSR/Cas9 sequence variants the *Fragaria PDS* gene, aligned to the reference (wild-type) sequence for exon 7 (120 base pairs). The wild-type (WT) nucleotide sequence is shown at the top of the chart and the positions of the 20 base pair target region ‘PDS74’ and PAM site (AGG) are indicated above the chart by dotted and solid lines, respectively. The ‘cut site’ is 3 base pairs upstream of the PAM site (AGG) and is shown as a vertical line. Each sequence variant type is identified by the location of the mutation relative to the cut site:number of bases deleted (D), inserted (I), or substituted (SNV:location relative to cut site and base substitution). Deleted bases generating gaps within exon 7 are shown by dashes. Base insertions and substitutions and consequent misaligned sequences relative to the wild type are highlighted. Sequences are shown truncated where mutations cause a frameshift generating stop codons within exon 7. **b** Amino acid sequences for each variant type aligned to the reference sequence (WT) for exon 7. Variant amino acids relative to the wild type are highlighted. Stop codons are shown in black and marked with an asterisk. Gaps in amino acid sequences within exon 7 are shown by a dashed line and partial amino acids are highlighted.
