# Supplementary material for: CRISPR/Cas9-mediated mutagenesis of phytoene desaturase in diploid and octoploid strawberry
Source: Plant Methods. 2019 May 2;15:45. doi: 10.1186/s13007-019-0428-6 (PMC6495592; doi:10.1186/s13007-019-0428-6)
Supplement: Supplementary file 8 — Additional file 8: Fig. S5. Gel electrophoresis of TAIL PCR amplification products for Calypso (a) and Hawaii 4 (b–d). [file 13007_2019_428_MOESM8_ESM.pdf]

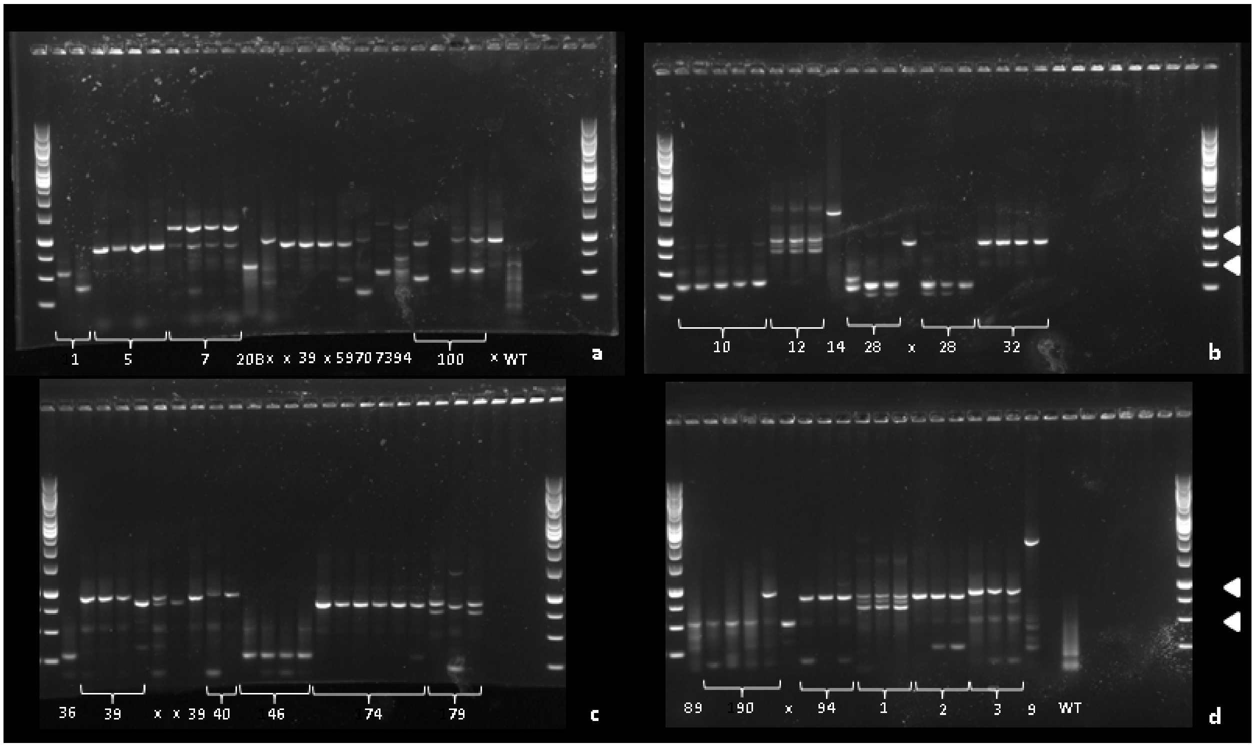

Fig. S5. Gel electrophoresis of TAIL PCR amplification products for Calypso (**a**) and Hawaii 4 (**b** - **d**).

Transgenic lines which were sequenced are numbered, samples within lines are in order according to Table S4. WT=wild-type, x = not sequenced. The arrows indicate 1 Kb and 500 bp ladder size markers.
