# Supplementary material for: CRISPR/Cas9-mediated mutagenesis of phytoene desaturase in diploid and octoploid strawberry
Source: Plant Methods. 2019 May 2;15:45. doi: 10.1186/s13007-019-0428-6 (PMC6495592; doi:10.1186/s13007-019-0428-6)
Supplement: Supplementary file 9 — Additional file 9: Fig. S6. Left border T-DNA integration. [file 13007_2019_428_MOESM9_ESM.pdf]

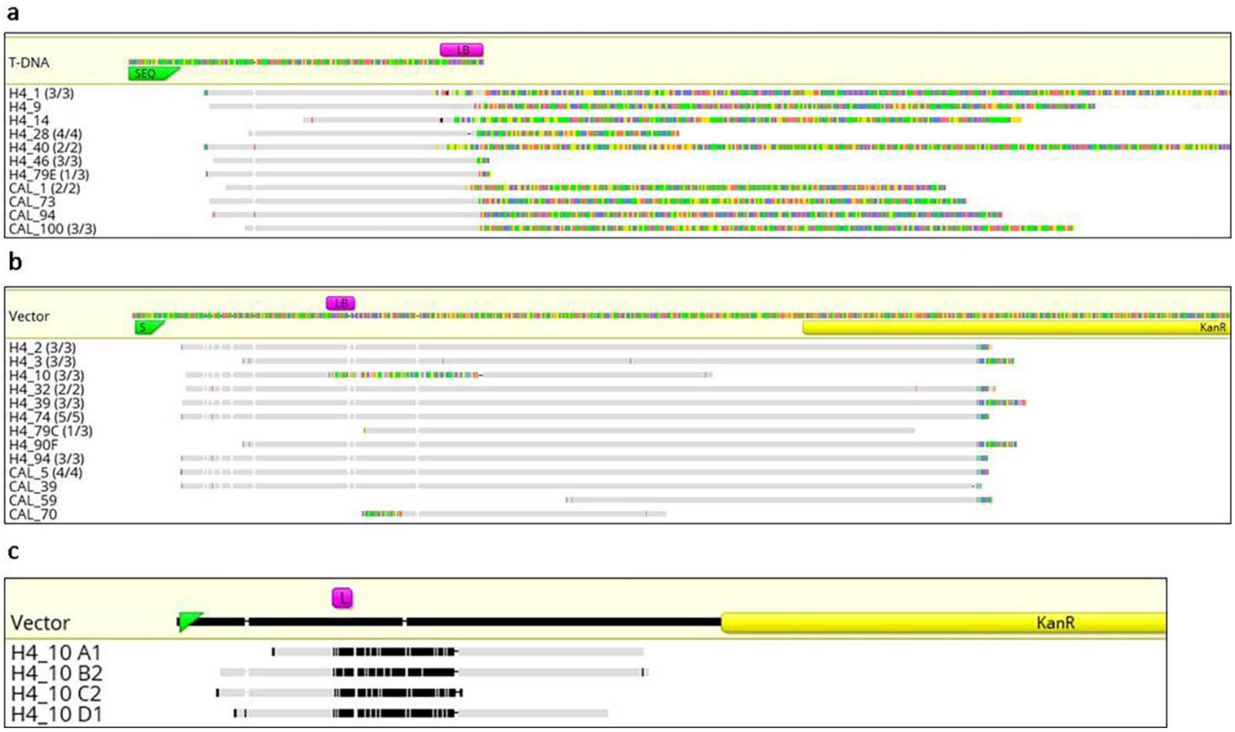

Fig. S6. Left border T-DNA integration. TAIL PCR sequence data for transgenic 'Hawaii 4' (H4) and 'Calypso' (Cal) lines.

a Assembly to the binary vector T-DNA. **b** Assembly to the binary vector pCas9-K-GFP. Where homologous data was obtained from more than one sample in a shoot line this is indicated (number of homologous shoots/number of shoots sequenced in shoot line) and sequence for only one (representative) sample is shown. **c** Assembly to T-DNA of sequence data for shoots from line 10. Mismatches to the reference sequences are highlighted. Mismatches are unique for each line. S/SEQ/green arrow=location of primer used for sequencing. Pink box labelled L/LB=position of T-DNA left border.
