# Supplementary material for: CRISPR/Cas9-mediated mutagenesis of phytoene desaturase in diploid and octoploid strawberry
Source: Plant Methods. 2019 May 2;15:45. doi: 10.1186/s13007-019-0428-6 (PMC6495592; doi:10.1186/s13007-019-0428-6)
Supplement: Supplementary file 10 — Additional file 10: Table S4. TAIL PCR amplicon sequences for transgenic shoot lines of ‘Hawaii 4’ and ‘Calypso’. [file 13007_2019_428_MOESM10_ESM.pdf]

'Hawaii 4'	
1 C	GGCTGAAGTCCAGCTGAAGTCCAGCTGCCAGAAACCCACGTCATGCCAGT
9 B	AAGACGTCGAAGAGCATAGCACGTCTCTTGAACAATTGCAACATTTTCAA
14	TTTACAACCTTCGGCTTCGGGTTCTGATTTGGGCCTGGTAACTGGTTTTT
28 C1	TCCTTTTCTACTGTCCTTTCGATGAAGTGACAGATAGCTGGGCAATGGAA
40 B	TGGGTGGAGGTTGCTGCCCAGACGTTCAAATCGATCTCGCCGGCAAGAGA
46 Diii	ATCCTTTGCTAC
79E	AGCCATG
'Calypso'	
1 A	GTAAGAATAAAATAAAATATATGGGCCACCGGCAGCAGTGAGAACACGCT
7 D	TTTTTTACCCTTTTTTATATTTTTTTTGTTCTCGATTTAATTTTAATGGT
73	TGCAAAGCAGCCGTAGTCCCTCTCCTTCGTGACTGTTACCAAAGGCAAAT
94	AGCACATCCCTCCTTCATTGTCATCATGAATCAAACAAATGTTTGTTCTT
100 2	GAATATGAAATATACCCTAGCTTGGATCTGATAAATTATCAGATCCGAGC

Table S4. TAIL PCR amplicon sequences for transgenic shoot lines of 'Hawaii 4' and 'Calypso'.

The sequences shown represent putative plant chromosomal sequences at the T-DNA integration site for representative shoots from each shoot line (where sequence information was obtained from multiple shoots). For sequences exceeding 50 bases only the first 50 bases are shown.
