# Supplementary material for: CRISPR/Cas9-mediated mutagenesis of phytoene desaturase in diploid and octoploid strawberry
Source: Plant Methods. 2019 May 2;15:45. doi: 10.1186/s13007-019-0428-6 (PMC6495592; doi:10.1186/s13007-019-0428-6)
Supplement: Supplementary file 11 — Additional file 11: Fig. S7. Amino acid sequences for published plant PDS genes aligned against Fragaria vesca. [file 13007_2019_428_MOESM11_ESM.pdf]

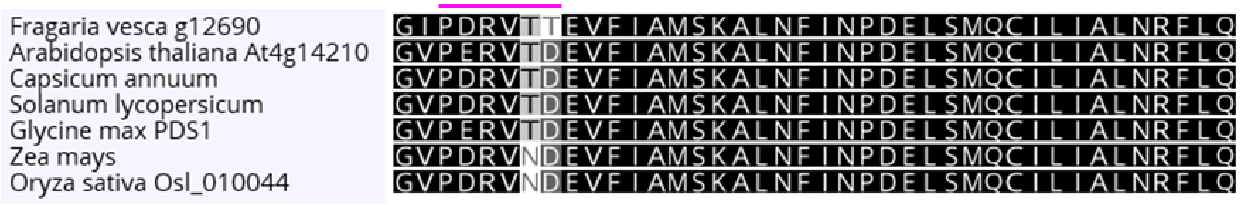

Fig. S7. Amino acid sequences for published plant *PDS* genes aligned against *Fragaria vesca.*

The extraction shown is the alignment for exon 7 of *F. vesca* g12690 (Fv assembly v4). The solid line above the alignment indicates the position of the CRISPR/Cas9 edited nucleotide target sequence.
