# Supplementary material for: CRISPR/Cas9-mediated mutagenesis of phytoene desaturase in diploid and octoploid strawberry
Source: Plant Methods. 2019 May 2;15:45. doi: 10.1186/s13007-019-0428-6 (PMC6495592; doi:10.1186/s13007-019-0428-6)
Supplement: Supplementary file 12 — Additional file 12: Table S5. Primer sequences. [file 13007_2019_428_MOESM12_ESM.pdf]

Name	Sequence
P5-300 F	*TCGTCGGCAGCGTCAGATGTGTATAAGAGACAG*TGAAGGGCTATTAGAAAATGAACAGT
P7-700 R	*GTCTCGTGGGCTCGGAGATGTGTATAAGAGACAG*TTTAACCATTCCAAAACTCCTTCTGG
TAIL R1	CATGCTCCTGTCAGAAATTCCGTGATCTTA
TAIL R2	CGGCCAATATCCTAAATGTGCG
TAIL SEQ	ATCCTAAATGTGCGTGGCTTTATCTGTCTT
AD3	AGWGNAGWANCAWAGG
mcs_F	AAGTCGTTAATGGCTGCGGA
mcs_R	GCCATCGCTACCTTAGGACC
Nos-Kan F	GATATCGATACATGAGAATTAAGGGAG
Nos-Kan R	GATATCAGCTTGCATGCCGG

Table S5. Primer sequences
5’ extensions are shown in italic font. Degenerate primers: W=A/T; S=G/C; N=A/T/G/C
