# Supplementary material for: CRISPR/Cas9-mediated mutagenesis of phytoene desaturase in diploid and octoploid strawberry
Source: Plant Methods. 2019 May 2;15:45. doi: 10.1186/s13007-019-0428-6 (PMC6495592; doi:10.1186/s13007-019-0428-6)
Supplement: Supplementary file 13 — Additional file 13: Table S6. PCR cycles used for analysis of plant samples. [file 13007_2019_428_MOESM13_ESM.pdf]

1. <u>TAIL PCR</u>

TAIL PCR first round

Stage 1 (x1 cycle)	95 ^{0}C 3 min
Stage 2 (x5 cycles)	95 ^{0}C 30 sec, 62 ^{0}C 1 min, 72 ^{0}C 30 sec
Stage 3 (x1 cycle)	95 ^{0}C 3 sec, 25 ^{0}C 3 min, * 72 ^{0}C 30 sec
Stage 4 (x15 cycles)	95 ^{0}C 10 sec, 68 ^{0}C 1 min, 72 ^{0}C 30 sec
	95 ^{0}C 10 sec, 68 ^{0}C 1 min, 72 ^{0}C 30 sec
	95 ^{0}C 10 sec, 44 ^{0}C 1 min, 72 ^{0}C 30 sec
Stage 5 (x1 cycle)	72 ^{0}C 5 min

*10% ramp (~0.34 ^{0}C per second)

TAIL PCR second round

Stage 1 (x1 cycle)	95 ^{0}C 3 min x1 cycle
Stage 2 (x5 cycles)	95 ^{0}C 10 sec, 64 ^{0}C 1 min, 72 ^{0}C 30 sec
Stage 3 (x15 cycles)	95 ^{0}C 10 sec, * 64 ^{0}C 1 min, * 72 ^{0}C 30 sec
	95 ^{0}C 10 sec, * 64 ^{0}C 1 min, * 72 ^{0}C 30 sec
	95 ^{0}C 10 sec, * 44 ^{0}C 1 min, * 72 ^{0}C 30 sec
Stage 4 (x5 cycles)	95 ^{0}C 10 sec, 44 ^{0}C 1 min, 72 ^{0}C 2 min, 72 ^{0}C 30 sec
Stage 5 (x1 cycle)	72 ^{0}C 5 min

*30% ramp (~1 ^{0}C per second),

Adapted from Liu et al (1995)

2. <u>Amplification of sgRNA insert in T-DNA</u>

Stage 1 (x1 cycle)	95 ^{0}C 5 min
Stage 2 (x35 cycles)	95 ^{0}C 30 sec, 52 ^{0}C 15 sec, 72 ^{0}C 10 sec
Stage 3 (x1 cycle)	72 ^{0}C 5 min

3. <u>Amplification of Nos-Kan insert in T-DNA</u>

Stage 1 (x1 cycle)	95 ^{0}C 5 min
Stage 2 (x35 cycles)	95 ^{0}C 30 sec, 46 ^{0}C 15 sec, 72 ^{0}C 30 sec
Stage 3 (x1 cycle)	72 ^{0}C 5 min

4. <u>Amplification of amplicons for NGS sequencing</u>

Stage 1 (x1 cycle)	98 ^{0}C 30 sec
Stage 2 (x35 cycles)	98 ^{0}C 10 sec, 65 ^{0}C 30 sec, 72 ^{0}C 12 sec
Stage 3 (x1 cycle)	72 ^{0}C 2 min

Table S6. PCR cycles used for analysis of plant samples. Reaction volumes: 1=20 μl; 2,3 = 25 μl; 4 = 50 μl.
